# Identification of the stress granule transcriptome via RNA-editing in single cells and *in vivo*

**DOI:** 10.1016/j.crmeth.2022.100235

**Published:** 2022-06-20

**Authors:** Wessel van Leeuwen, Michael VanInsberghe, Nico Battich, Fredrik Salmén, Alexander van Oudenaarden, Catherine Rabouille

**Affiliations:** 1 Hubrecht Institute of the KNAW & UMC Utrecht, Utrecht, the Netherlands; 2 Section Cell Biology, Center for Molecular Medicine, University Medical Center Utrecht, Utrecht, the Netherlands; 3 Department of Biomedical Sciences in Cells and Systems, UMC Groningen, Groningen, the Netherlands

**Keywords:** stress granules, RNA, RNA-editing, hyperTRIBE, S2 cells, single cell, neurons, *Drosophila*, VASA-seq

## Abstract

Stress granules are phase-separated assemblies formed around RNAs. So far, the techniques available to identify these RNAs are not suitable for single cells and small tissues displaying cell heterogeneity. Here, we used TRIBE (target of RNA-binding proteins identified by editing) to profile stress granule RNAs. We used an RNA-binding protein (FMR1) fused to the catalytic domain of an RNA-editing enzyme (ADAR), which coalesces into stress granules upon oxidative stress. RNAs colocalized with this fusion are edited, producing mutations that are detectable by VASA sequencing. Using single-molecule FISH, we validated that this purification-free method can reliably identify stress granule RNAs in bulk and single S2 cells and in *Drosophila* neurons. Similar to mammalian cells, we find that stress granule mRNAs encode ATP binding, cell cycle, and transcription factors. This method opens the possibility to identify stress granule RNAs and other RNA-based assemblies in other single cells and tissues.

## Highlights

• Identification of stress granule (SG) RNAs using the RNA-editing method hyperTRIBE
• FMR1-ADAR fusion targets editing of SG transcripts after arsenite treatment
• Application to single and bulk *Drosophila* S2 cells and neurons
• Suitable for single-cell profiling of SG RNAs from small amounts of tissue

## Motivation

Sequencing-based methods for identifying stress granule RNAs (Khong et al., 2017; Namkoong et al., 2018; Padrón et al., 2019) have typically required large amounts of input material and can therefore be challenging for single-cell studies on low-volume, heterogeneous tissues. To address this limitation, we have developed an application of hyperTRIBE that allows the identification of stress granule RNAs in single cells and *Drosophila* neurons.

## Graphical abstract

**Figure:**
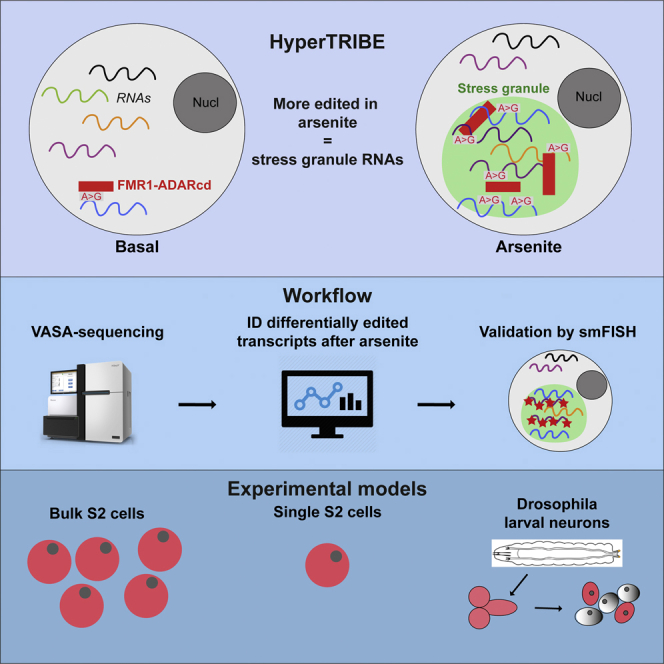

## Teaser

van Leeuwen et al. adapt an RNA-editing method to identify the stress granule (SG) transcriptome of bulk and single *Drosophila* cells. Fusion of the editing enzyme to FMR1 targets editing of SG transcripts, which are identified via VASA-seq. SG transcripts are involved in ATP binding, cell cycle, and transcriptional regulation.

## Introduction

Non-membrane-bound compartments represent an important aspect of cell organization. They are formed by phase separation, where solution of seemingly diffuse macromolecules segregates into two distinct phases (Hyman et al., 2014; Gomes and Shorter, 2018). Interestingly, cellular stress induces the formation of many of these compartments (van Leeuwen and Rabouille, 2019), including stress granules (Anderson and Kedersha, 2002; Aulas et al., 2017) that formed around mRNAs after cells have been subjected to many cellular stresses.

The protein content of stress granules has been extensively studied (Jain et al., 2016; Youn et al., 2019; Markmiller et al., 2019; Aulas et al., 2017). The biophysical principles underlying stress granule phase separation has also been elucidated *in vitro* using purified RNA-binding proteins (RBPs) and their RNA binding domains (Patel et al., 2015; Murray et al., 2017; Molliex et al., 2015).

Recently, RNAs have also been shown to be structural components of stress granules (Zhang et al., 2015; Van Treeck et al., 2018) both *in vitro* and *in vivo*. This is sustained by the demonstration that elements in RNA secondary structure are conducive to phase separation (Langdon et al., 2018). This renders the identification of stress granule RNAs an important biological question.

This has recently been performed in mammalian cultured cells after stress granule core isolation (Namkoong et al., 2018; Khong et al., 2017), cross-linking and immunoprecipitation (Anders et al., 2018), and proximity biotinylation of RNAs (Padrón et al., 2019). These studies revealed that stress granules mainly harbor longer mRNAs that are thought to be more poorly translated by ribosomes. Furthermore, even though a clear enrichment in adenylate-uridylate-rich elements (AREs) has been established in endoplasmic reticulum-stress driven stress granule mRNAs (Namkoong et al., 2018), a true recruitment motif has yet to be identified, although recent efforts show that AREs and Pumilio recognition elements increase mRNA partitioning into stress granules (Matheny et al., 2021).

However valuable, these techniques are somewhat limited by the step of cell fractionation and stress granule purification or by RNA pulldown. Consequently, they require a substantial amount of input materials (Wheeler et al., 2016; Jain et al., 2016), thus preventing the analysis of limited/small tissues and single cells. This represents a critical step, as there are now indications that the RNA content of stress granules might be heterogeneous within a cell population (Khong et al., 2017). Furthermore, it might be important to properly compare the differential response to stress of a given cell in its environment (Aulas et al., 2017).

Here, we adapted a method to identify RNAs that are recruited to stress granules, which does not require fractionation and isolation, and that works in single cells and in tissue in a cell-type-specific manner. This method is based on TRIBE (Target of RNA-binding proteins Identified by Editing) first established by the Rosbash group (McMahon et al., 2016). There, a given RBP is fused to the catalytic domain of the DNA/RNA-editing enzyme ADAR (ADARcd) that deaminates adenosine-to-inosine on RNA molecules, which is read out as A-to-G mutations by sequencing. This method has been used to successfully identify the RNA targets of several RPBs in *Drosophila* neurons (McMahon et al., 2016). An activating mutation in ADARcd (TRIBE) has since been shown to increase the editing efficiency of ADARcd by 400% when compared with the original (Xu et al., 2018). The purification-free method we adapted from TRIBE enables the identification of stress granule RNAs in single tissue cultured cells, and in specific tissues, the neurons of the *Drosophila* larval brain.

## Results

### Predictions and experimental setup

**Figure 1:**
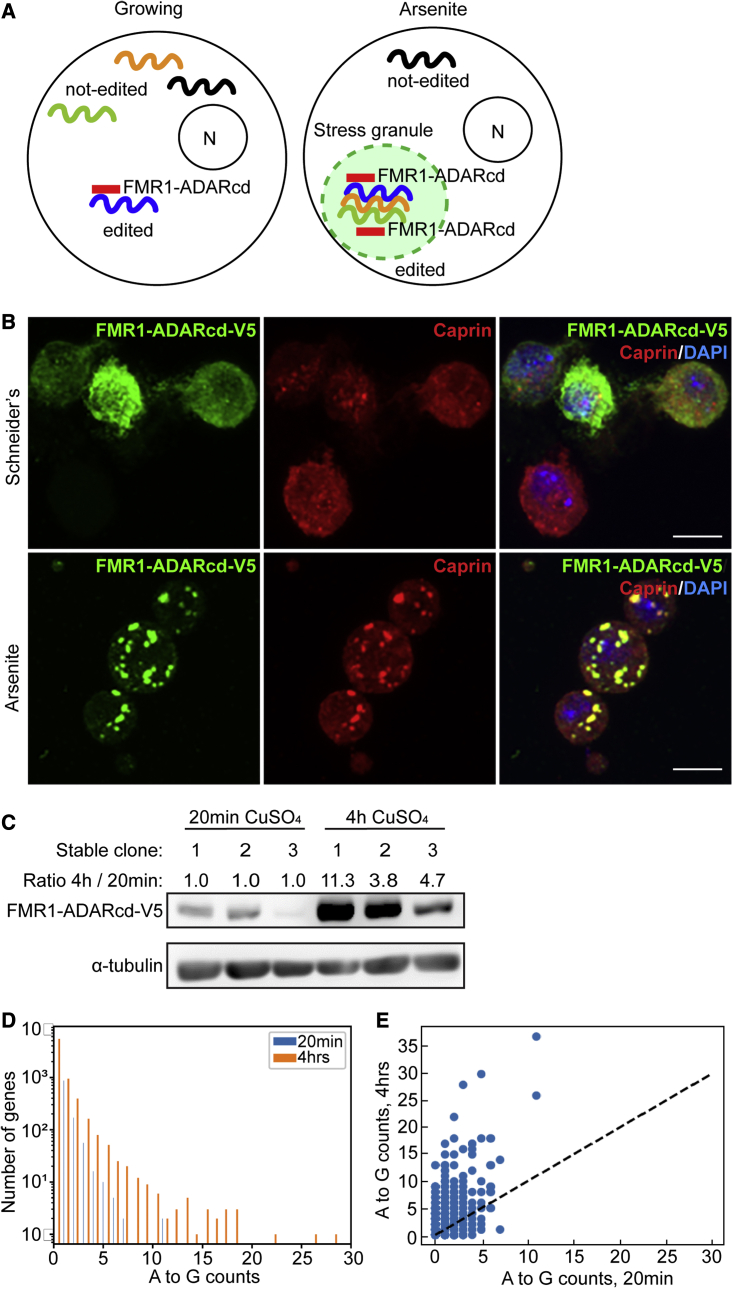
Adaptation of HyperTRIBE using FMR1-ADARcd-V5 to detect stress granule RNAs (A) Schematic overview of our HyperTRIBE adaptation strategy using FMR1-ADARcd-V5 to predict stress granule RNAs. In growing cells, FMR1-ADARcd-V5 binds to its own client RNAs that can be edited in the cytosol. Upon arsenite stress, FMR1-ADARcd-V5 is recruited to stress granules. Allowing ADARcd to edit stress granule RNAs, its clients as well as other stress granule RNAs that are in close proximity. (B) Immunofluorescence visualization of FMR1-ADARcd-V5 (using anti-V5, green) in S2 cells in Schneider’s and in arsenite (0.5 mM for 4 h) leading to localization of FMR1-ADARcd-V5 with the known stress granule marker Caprin (red). (C) Western blot of S2 cell extract after 20 min and 4 h induction (+CuSO4) of different clones expressing FMR1-ADARcd-V5 using an anti-V5 antibody. This number indicated is the ratio “4 h/20 min induction” of the ratio “FMR1-ADARcd-V5/tubulin” for each clone (20 min is therefore 1). (D and E) Graph displaying the frequency (D) and total (E) of A>G editing events in S2 cells in which the expression of FMR1-ADARcd-V5 was induced for 20 min and 4 h. Scale bar: 10 μm (B).

To identify the RNA content in stress granules, we adapted the TRIBE method (Xu et al., 2018; McMahon et al., 2016) in such a way that most of the RNA editing takes place in stress granules. The prediction is that the more an RNA is found specifically edited in stress conditions (here arsenite treatment), the more likely it is recruited in stress granules (Figure 1A). As in TRIBE (Xu et al., 2018), we used the RBP FMR1 tagged to ADARcd-V5 (FMR1-ADARcd-V5). Endogenous (Aguilera-Gomez et al., 2017) and GFP-tagged FMR1 (Zacharogianni et al., 2014) are efficiently recruited to stress granules on arsenite stress. FXR1, the mammalian homolog of *Drosophila* FMR1, is largely immobile within stress granules (Marmor-Kollet et al., 2020), in a similar manner to FMR1 (Zacharogianni et al., 2014), potentially reducing its dynamics in and out of the stress granules. Last, fusing ADARcd to FMR1 did not appear to disrupt FMR1 function and its binding to other RBPs and RNAs (Xu et al., 2018). In addition, ADARcd remained functional when fused to FMR1 (Xu et al., 2018).

These edited RNAs (predicted to be in stress granules) will likely include FMR1 clients, but we hypothesize that they will also include RNAs that are clients of other stress granule RBPs that would then be edited in *trans*.

To achieve this, we first verified that FMR1-ADARcd-V5 is indeed efficiently recruited in arsenite-induced stress granules. S2 cell clones stably expressing FMR1-ADARcd-V5 showed its 8.3 ± 2.6-fold concentration in foci also positive for the RBP Caprin, a known marker of stress granules, in 90% of the cells (Figures 1B and S1A, *A’*). Arsenite treatment of S2 cells for 4 h is longer than normally used for mammalian cells but we show that it does not result in cell death. S2 cells are still viable after this treatment and grow again when the stress is removed, despite a loss of 20% (Figure S1B). Interestingly, stress granule dissolution occurs before cells start growing again (Figures S1B and S1C), suggesting that stress granule dissolution precedes growth recovery after stress relief.

Second, to detect a significant differential RNA editing in stress conditions, the RNA editing in basal conditions (non-stressed cells during which FMR1-ADARcd-V5 can edit FMR1 clients independently of their further recruitment in stress granules) should be kept as low as possible. The notion was to find a tradeoff in producing enough of chimeric protein to lead to RNA editing in stress granules without generating a high background in non-stressed conditions. To do so, we expressed FMR1-ADARcd-V5 (which is under control of a copper sulfate inducible metallothionein promoter) for 20 min and 4 h before applying the stress. Four hours of induction of clone 1 resulted in 11.3-fold higher FMR1-ADARcd-V5 expression compared with 20 min (Figure 1C).

Last, we verified that the induction of FMR1-ADARcd-V5 in clone 1 is able to produce detectable editing on transcripts. To detect these, we used a variant caller that locates specific base changes on the transcripts (McKenna et al., 2010) (see method details section, and below). We identified 1,727 RNAs displaying edited bases. As expected, most of the filtered *de novo* variants detected were A-to-G events (Figure 1D), while other possible nucleotide changes were hardly detected (Figures S2A*–*S2C). Since the levels of RNA editing after a 4 h induction showed a clear increase when compared with 20 min (Figures 1D and 1E), we chose a 4 h induction of clone 1 in the rest of the experiments.

### RNA editing through ADAR predicts the stress granule transcriptome in S2 cells

**Figure 2:**
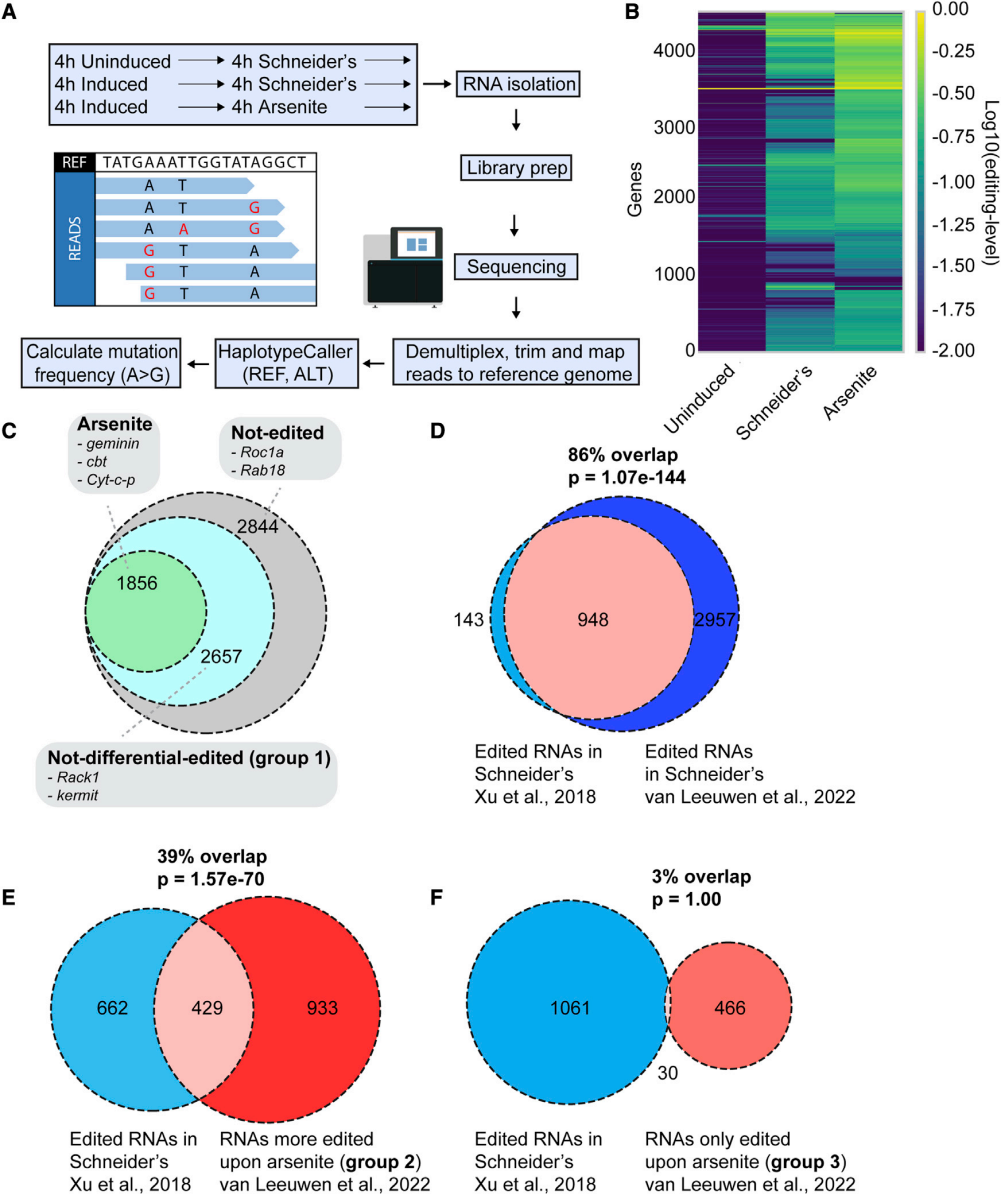
RNA editing through ADAR predicts the stress granule transcriptome in S2 cells (A) Schematic overview of the workflow. After induction of the FMR1-ADARcd-V5 expression for 4 h in Schneider’s, cells were either maintained in Schneider’s or treated with 0.5 mM arsenite for 4 h. RNA was isolated, and libraries were generated and sequenced. Reads were processed by demultiplexing, trimming and mapping to the reference genome. The mapped reads were consequently subjected to a haplotype caller to detect base editing events (see Figure S4). The frequency of A>G editing events is calculated per gene, per sample and per condition. (B) Heatmap displaying the editing level per gene and per condition. (C) Venn diagram depicting the number of RNAs that were significantly (p < 0.01) more edited upon arsenite after applying the Empirical Bayes statistical test. (D) Venn diagram depicting the percentage of RNAs that are edited in Schneider’s and that overlap with the established FMR1 clients (1,091) (Xu et al., 2018). The overlap (p value) was calculated using a Hypergeometric test. (E and F) Venn diagram depicting the percentage of RNAs that were more edited in Schneider’s and more edited upon arsenite (group 2, E), RNAs that were only edited upon arsenite (group 3, F) and that overlap with the established FMR1 clients (Xu et al., 2018).

To identify RNAs present in stress granules, we used the setup conditions described above (4 h induction in basal condition, i.e., Schneider’s) followed by cell incubation with arsenite for 4 h (Figure 2A). Control cells were maintained in Schneider’s for the same length of time. An uninduced sample was also included as a control for endogenous editing. Total RNAs from triplicate experiments were generated and sequenced using the new sequencing method VASA-seq (Hollfelder et al., 2022). VASA-seq does not rely on primers binding to the 3′ or 5′ end of the transcripts. It allows the sequencing of the full transcript and permits the detection of mutations throughout the RNA sequence. This is critical, as editing by ADAR might not be limited to the 3′ and 5′ UTR due to FMR1 binding its RNA clients mainly in their coding region (Li et al., 2020).

Mapping the reads (read depth of 1∗10^7^ reads) to the *Drosophila* genome (Figure 2A) revealed that over all conditions 7,357 RNAs were detected with an expression level higher than one transcript per million. Lower expression was deemed too low and those were removed. Pairwise correlation analysis of the expression level of the (total and edited) transcriptome between samples and within triplicates revealed proper clustering (Figures S3A and S3B), indicating a high reproducibility between libraries.

We noticed FMR1-ADARcd edits at select sites rather than uniformly across a given transcript. When comparing the conditions (uninduced, Schneider’s, and arsenite), the same adenosine appear to be edited. For example, along the 500-nt region in the *pzg* transcript, only five of the 147 adenosines exhibit an increase in mutation frequency between the conditions (vertical lines, Figure S4A). This non-randomness was observed for many of the edited transcripts and was confirmed by a genome wide examination of editing across conditions. This reveals that specific adenosines are moderately but specifically edited (Figure S4B).

Consequently, in order to determine the identity of the specific edited RNAs as well as the level of their editing, we used a variant caller (GATK [McKenna et al., 2010]). For each of these specific edited positions, we extracted the total number of mutated bases in each sample in triplicate, and used these to calculate the editing frequency (e.g., 17 of the 53 bases at position 1 are “G,” giving a mutation frequency of 0.32 for arsenite) (Figure S4C). The editing frequency was calculated for each specific position across all conditions. All identified variant positions and their corresponding editing frequencies for a gene were then averaged (“average editing frequency”) to compare the average editing frequency between conditions (Figure S4C). Note that the calculation of the editing frequency could be done using alternative strategies (please see more in the limitations of the study and STAR Methods).

Using this workflow (Figure S4C), we first determined the level of endogenous editing by comparing the uninduced samples with those maintained in basal condition (Schneider’s). The Euclidean distance between samples was then calculated and visualized. In total, we found 2,844 non-edited RNAs and 4,513 FMR1-ADARcd edited RNAs, including 905 RNAs endogenously edited at a low level (Figures 2B, Table S1, Figures S4A and S4B).

When comparing samples in Schneider’s with those treated with arsenite, we found that, as predicted, the editing extent was higher in arsenite stress than in Schneider’s (Figures 2B, S4A, and S4B). In addition, some genes appear only edited in arsenite and not in Schneider’s (Figure 2B). To identify the RNAs only or more edited upon arsenite stress, we set that the average editing frequency of RNAs from the “arsenite” triplicates should be significantly higher than the average editing frequency in Schneider’s (basal condition) (p < 0.01) using the Empirical Bayes test. Using this definition, 2,657 RNAs (group 1), such as *Rack1* and *kermit*, were found not to be differentially edited (Figures 2C and Table S1).

Conversely, 1,856 RNAs were found to be more edited upon arsenite when compared with Schneider’s (Figures 2B and 2C and Table S1, see also alternative strategy in STAR Methods). These were divided into two groups. Group 2 contains 1,362 RNAs (73%), such as *row*, *Cyt-c-p*, *red*, *cbt*, and *Khc*, that were also edited at low level in basal conditions. Conversely, group 3 (496 RNAs, 27%), which includes *geminin* and *Prosβ4*, were only edited under arsenite stress. According to our predictions, groups 2 and 3 RNAs are predicted to be enriched in stress granules (see below).

We then compared the RNAs we found edited in Schneider’s with the list of FMR1 clients established by the Rosbash group (Xu et al., 2018), using a geometric test. The overlap was 86%, showing the reliability of our method in detecting FMR1-ADARcd editing (Figure 2D).

We then assessed whether the RNAs of groups 2 and 3 are FMR1 clients. As above, we compared these RNAs with the list of FMR1 clients established by the Rosbash group (Xu et al., 2018). Group 2 RNAs (edited in Schneider’s but more edited in arsenite) present a 39% overlap (429 RNAs) with these clients (Figure 2E, Table S2). Group 3 RNAs (only edited only upon arsenite) overlap with the list of the FMR1 clients by 3% (30 RNAs) (Figure 2F and Table S2). Altogether, only 25% (459 of the 1,856 RNAs) are FMR1 clients.

Taken together, these results show that we have potentially identified 1,856 RNAs that recruited to stress granules upon arsenite stress. Furthermore, the stress granule RNAs we retrieved do not appear to be simply FMR1 clients that are passively bound to FMR1-ADARcd upon its recruitment to the stress granules.

### Validation of identified stress granule transcriptome by smFISH

**Figure 3:**
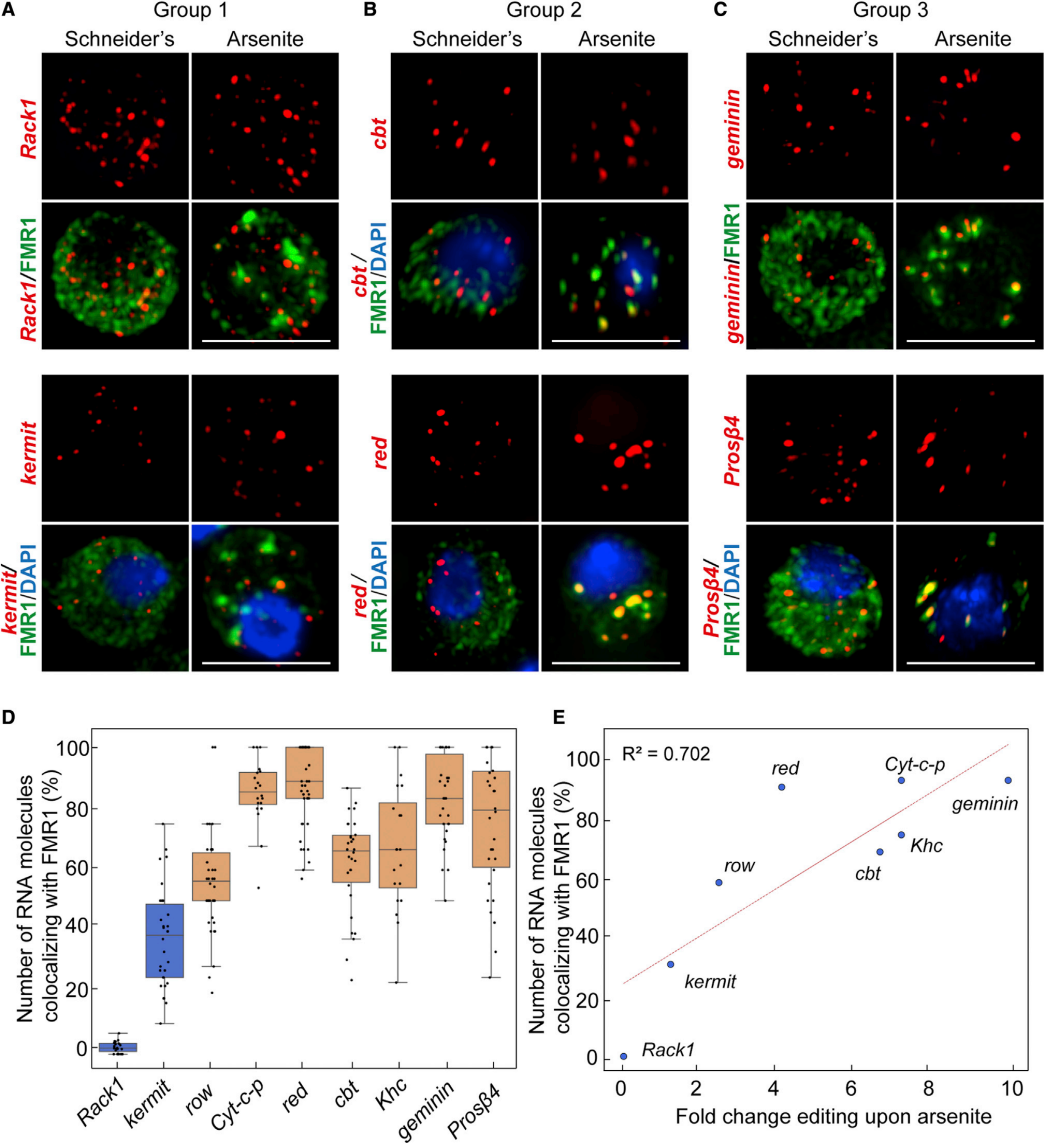
Potential stress granule RNAs identified by RNA editing are validated by single-molecule FISH (A) Visualization by smFISH of *Rack1* and *kermit* mRNAs (group 1). (B) Visualization by smFISH of *cbt* and *red* mRNAs (group 2). (C) Visualization by smFISH of *geminin* and *Prosβ4* mRNAs (group 3). (D) Quantification of the number per cell of RNA molecules in stress granules (colocalization with FMR1). (E) Scatterplot displaying the number of RNA molecules in stress granules (FMR1 positive) versus the fold change editing upon arsenite compared to Schneider’s. Scale bar: 10 μm (A, B, C).

To validate our prediction, i.e., to show that group 2 and 3 RNAs are indeed localized and enriched to arsenite-induced stress granules, we used single-molecule fluorescence *in situ* hybridization (smFISH). Group 1 RNAs (such as *Kermit* and *Rack1)* are non-differentially edited upon arsenite, and are therefore predicted not to be enriched in stress granules. Indeed, only 2% of *Rack1* RNA and 29% of *kermit* RNA overlap with stress granules (Figures 3A and 3D). Interestingly, *Rack1* is a clear FMR1 client (Xu et al., 2018). Yet, *Rack1* is not recruited in stress granules upon coalescence of its RBP.

Conversely, smFISH analysis revealed that RNAs of group 2 (such as *row*, *Cyt-c-p*, *red*, *cbt*, and *Khc*) and of group 3 (*geminin* and *Prosβ4*) all strongly co-localize (from 54% to 83%) with endogenous FMR1 in stress granules (Figures 3B, 3C, and 3D), validating their enrichment in stress granules.

Furthermore, we established a significant relationship between the fold change in editing frequency of a given RNA (between arsenite and Schneider’s) and the fraction of these RNA molecules localized in stress granules (quantified by smFISH). We observed an increased fold in editing that positively correlates with the localization in stress granules (*R*^2^ = 0.702, Figure 3E). For example, *cbt* is edited 6.7-fold higher in arsenite when compared with Schneider’s, and 62% of *cbt* RNA molecules localize to stress granules. Conversely, the fold change in editing for *Rack1* is 0.7 and the fraction of *Rack1* RNAs in stress granules is 2%. This strong correlation indicates that the higher the fold change in editing the most likely RNAs localize in stress granules.

Overall, the successful smFISH validation of these 9 RNAs (all chosen randomly, except *Rack1*) in stress granules suggests that the 1,856 RNAs that are differentially edited upon arsenite are potentially enriched in stress granules (see discussion).

### Arsenite-induced stress granules contain long mRNAs that encode ATP binding, transcription, cell cycle, and splicing factors

**Figure 4:**
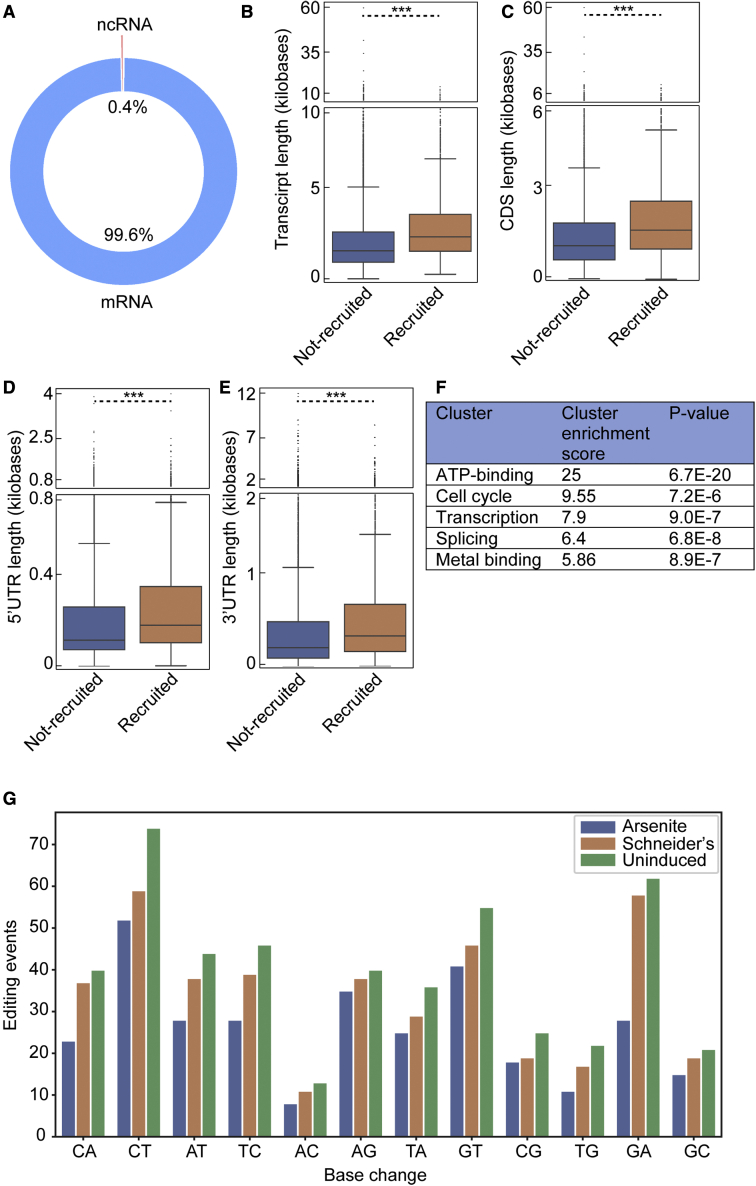
Features of the identified stress granule RNAs (A) Chart depicting the types of RNAs predicted to be in stress granules. (B–E) Boxplots displaying the length of transcripts (B), CDS (C), 5′UTRs (D), and 3′UTRs (E) of the mRNAs predicted to be in stress granules (recruited) and of the RNAs that were predicted not recruited. ∗∗∗p value < 0.001 (Mann-Whitney *U* test). (F) Gene enrichment analysis (using DAVID) of the RNAs predicted to be localized in stress granules upon arsenite. (G) Editing events per possible base change in intronic RNA sequences of bulk S2 cells.

We then asked whether the 1,856 predicted stress granule RNAs have common and specific features. First, we found that ∼99.6% are mRNAs and ∼0.4% are non-coding RNAs (ncRNAs) (Figure 4A). The stress granule transcripts are 1.3-fold longer than the mRNAs predicted not to be in stress granules (Figure 4B), an increase that is not specific to the coding sequence (CDS), 5′ UTR, and 3′ UTR (Figures 4C–4E). Finally, a gene ontology (GO) analysis on the stress granule mRNAs showed a gene enrichment in ATP binding (kinases, chaperones, and cytoskeleton), transcription factor, RNA splicing, and cell cycle factors (Figure 4F).

Since VASA-seq is able to detect pre-mRNAs, we use the same variant caller approach as above on intronic RNA sequences. We found a low level of endogenous editing of these sequences. We also did not observe a significant increase in A-to-G editing between the uninduced and Schneider’s (Figure 4G), indicating that pre-mRNAs are not edited by FMR1-ADARcd-V5 in growing conditions. Moreover, A-to-G base changes were not dominantly present when compared with all other nucleotide base changes. Specific A-to-G editing also did not increase upon the arsenite condition. Therefore, we predict that pre-mRNAs are likely not present in stress granules in agreement with (Khong et al., 2017).

Together, these features suggest that stress granules may harbor and protect long mRNAs that encode proteins necessary for the cells to thrive once the stress is relieved.

### Identification of stress granule RNAs in single cells

There are clear indications that the RNA content of stress granules might be heterogeneous within a cell population (Khong et al., 2017). Our own smFISH data revealed that, overall, each cell does not have the same stress granule RNA enrichment as its neighbor (Figure S5A). The existing published techniques investigating the RNA content of stress granules require a substantial amount of material (see introduction), which makes single-cell analysis difficult. Given that our adapted hyperTRIBE technique does not require purification and pulldown, we tested the ability of this method (when combined with VASA-seq) (Hollfelder et al., 2022) to identify stress granule RNAs in single cells.

**Figure 5:**
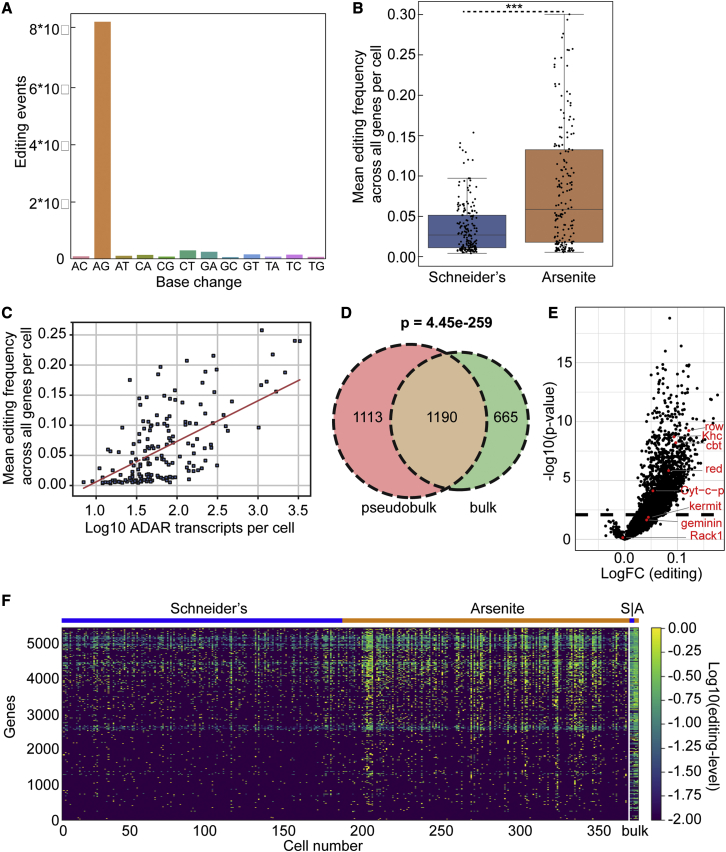
Identification of stress granule RNAs in single cells (A) Countplot displaying the number of editing events per possible base change in single cells. (B) Boxplot of the mean editing frequency of all genes per single cell for each condition. Each black dot represents a single cell. ∗∗∗p value < 0.001. (C) Scatterplot displaying the mean editing frequency (A-to-G) over all genes per single cell versus the number of ADAR transcripts per cell. (D) Venn diagram depicting the overlap of RNAs significantly (p < 0.01) more edited upon arsenite in bulk and in pseudobulk (pooled single cells). (E) Volcano plot depicting the log-fold change in editing and the significancy (p < 0.01, dashed line) for all detected RNAs in pseudobulk. (F) Heatmap of the editing level per gene per single cell (360 cells in total). The last column is from bulk sequencing (S = Schneider’s, A = arsenite).

The editing efficiency in bulk S2 cells was high enough to attempt detecting RNA editing in single cells. The same procedures as above resulted an average read depth of 70,000 reads per cell. After applying the variant caller, we observed a significant enrichment in A-to-G editing compared with all other possible nucleotide base changes (Figure 5A), indicating that hyperTRIBE can be used to detect RNA editing in single cells (pseudobulk of combined 360 analyzed single cells). As in bulk (see above), we determined the mean editing frequency per gene per cell. Interestingly, as in bulk (Figures 2B, S4A, and S4B), the editing activity upon arsenite treatment was found significantly higher when compared with Schneider’s (Figure 5B). Yet, not all single cells display high levels of editing. Some did not show editing, correlating with a very low expression of FMR1-ADARcd (Figure 5C).

In the pseudobulk, we identified 2,303 RNAs that were more edited upon arsenite, which show a significant overlap (64%) with the 1,856 RNAs found by bulk sequencing (Figure 5D). Notably, we found that most of the RNAs validated with smFISH to be enriched in stress granules were also significantly edited in pseudobulk, showing the reliability of the single-cell analysis (Figure 5E).

By comparing the editing frequency per gene in all cells, we observed the largest variation between conditions (Schneider’s and arsenite), not within a given condition. There, only a small degree of variation in the editing level between the cells is observed, suggesting a low level of heterogeneity (Figure 5F). Taken together, using our adaptation of the hyperTRIBE method, stress granule RNAs can be reliably identified in single cells, as they are similar to the stress granule RNAs found in bulk S2 cells.

### Identification of stress granule RNAs in *Drosophila* neurons

**Figure 6:**
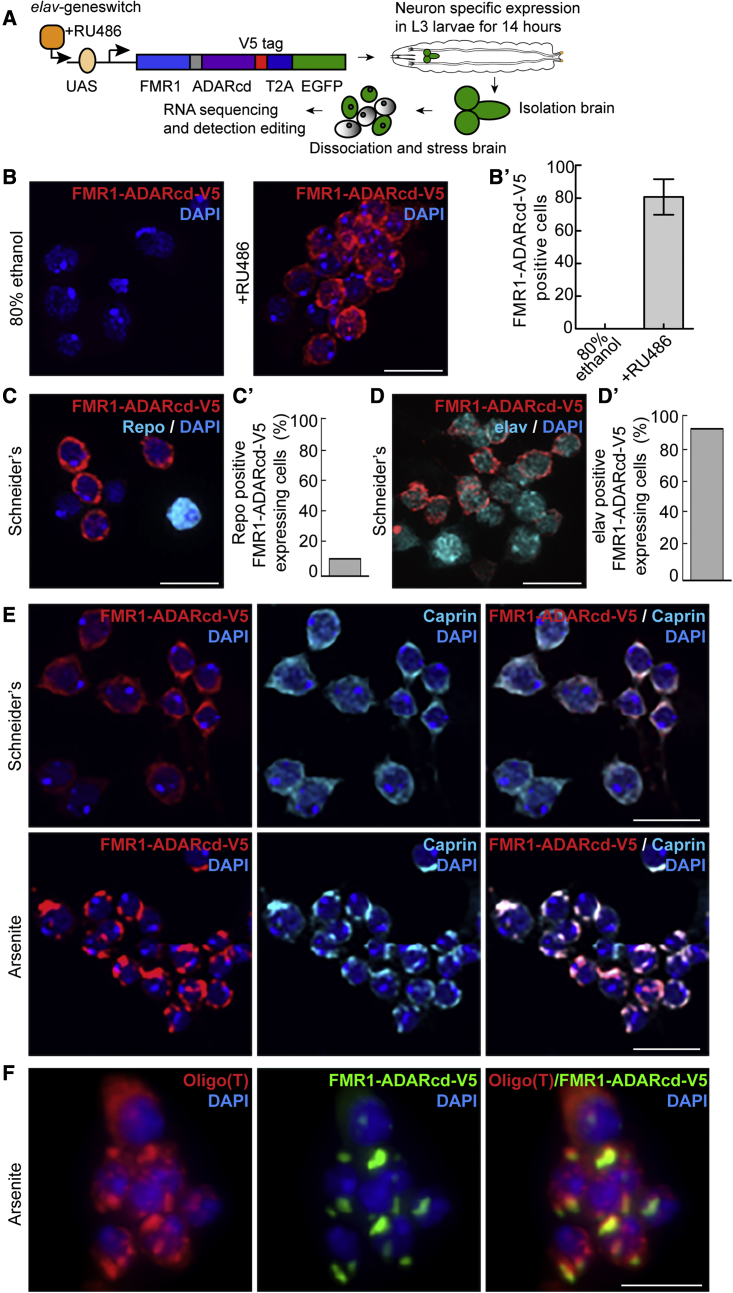
Stress granule formation in *Drosophila* neurons (A) Schematic overview of the workflow. *elav*-GeneSwitch-Gal4/FMR1-ADARcd-V5 expressing third instar larvae were exposed to RU486 by larval bathing to allow the expression of FMR1-ADARcd-V5 specifically in neurons. The dissociated brain cells were exposed to 0.5 mM arsenite for 4 h. Finally, the total RNA was isolated, sequenced, and the RNA editing analyzed. (B) Immunofluorescence visualization of FMR1-ADARcd-V5 (anti-V5, red) in dissociated *Drosophila* brain cells from mock (80% ethanol) and RU486 (3 mg/mL) bathed third instar larvae. Quantification in (B’). (C and D) Immunofluorescence visualization of FMR1-ADARcd-V5 (anti-V5, red), the glial cell marker Repo (cyan, C) and the neuronal marker Elav (cyan, D) in dissociated *Drosophila* brain cells. Quantification in (C’ and D’). (E) Immunofluorescence visualization of FMR1-ADARcd-V5 (anti-V5, red) in dissociated *Drosophila* brain cells upon incubation in Schneider’s and upon arsenite (0.5 mM for 4 h) leading to FMR1-ADARcd-V5 localization in stress granules together with Caprin (green). (F) Visualization of polyadenylated mRNAs (red) in arsenite-stressed (0.5 mM for 4 h) neurons by RNA FISH using an oligo(T) probe, in FMR1-ADARcd-V5 positive stress granules. Scale bar: 10 μm (B, C, D, E); 5 μm (F).

We then tested whether this hyperTRIBE-based method could be used to establish the RNA content of stress granules in specific cell types in primary tissues, and we focused on neurons of the *Drosophila* third instar larval brain. We sought to specifically express the FMR1-ADARcd-V5 fusion in *Drosophila* neurons, while allowing for precise temporal control to limit basal editing in unstressed conditions. To achieve this, we used the geneswitch/Gal4 system (Osterwalder et al., 2001) in which a modified Gal4/UAS system (Phelps and Brand, 1998) can lead to increased protein expression upon addition of the drug RU486 (here for 14 h). To limit the expression to neurons, we selected the pan-neuronal *elav*-geneswitch-Gal4. Thus, FMR1-ADARcd-V5 was specifically expressed in *Drosophila* neurons by bathing third instar larvae in RU486 (Figure 6A).

Dissection and dissociation of brains into isolated cells (neurons and glia) showed that 80% of them expressed FMR1-ADARcd-V5 (Figures 6B and 6B’), whereas there was no detectable expression in the absence of the drug. We confirmed that FMR1-ADARcd-V5 was solely expressed in neurons and not in glial cells. Indeed, FMR1-ADARcd-V5 was only expressed 9% in Repo positive (glial) cells (Figures 6C and 6C’), while 93% of the elav-positive cells did express FMR1-ADARcd-V5 (Figures 6D and 6D’). These data indicate that the *elav*-GeneSwitch-Gal4 driver allows the expression of FMR1-ADARcd-V5 specifically in neurons. Last, we tested whether dissociated neurons form FMR1-ADARcd-V5 positive stress granules upon arsenite treatment. Ninety-one percent of the dissociated neurons form stress granules (Caprin positive) very efficiently (i.e., they form at least one stress granule per cell) upon 4 h incubation with arsenite (Figure 6E). As expected, these stress granules contain polyadenylated RNAs as shown by RNA FISH using an oligo(dT) probe (Figure 6F). These results show that when stressed by arsenite, larva brain neurons form *bona fide* stress granules where FMR1-ADARcd-V5 is recruited, as in S2 cells.

### RNA editing through ADAR predicts the stress granule transcriptome of dissociated *Drosophila* neurons

**Figure 7:**
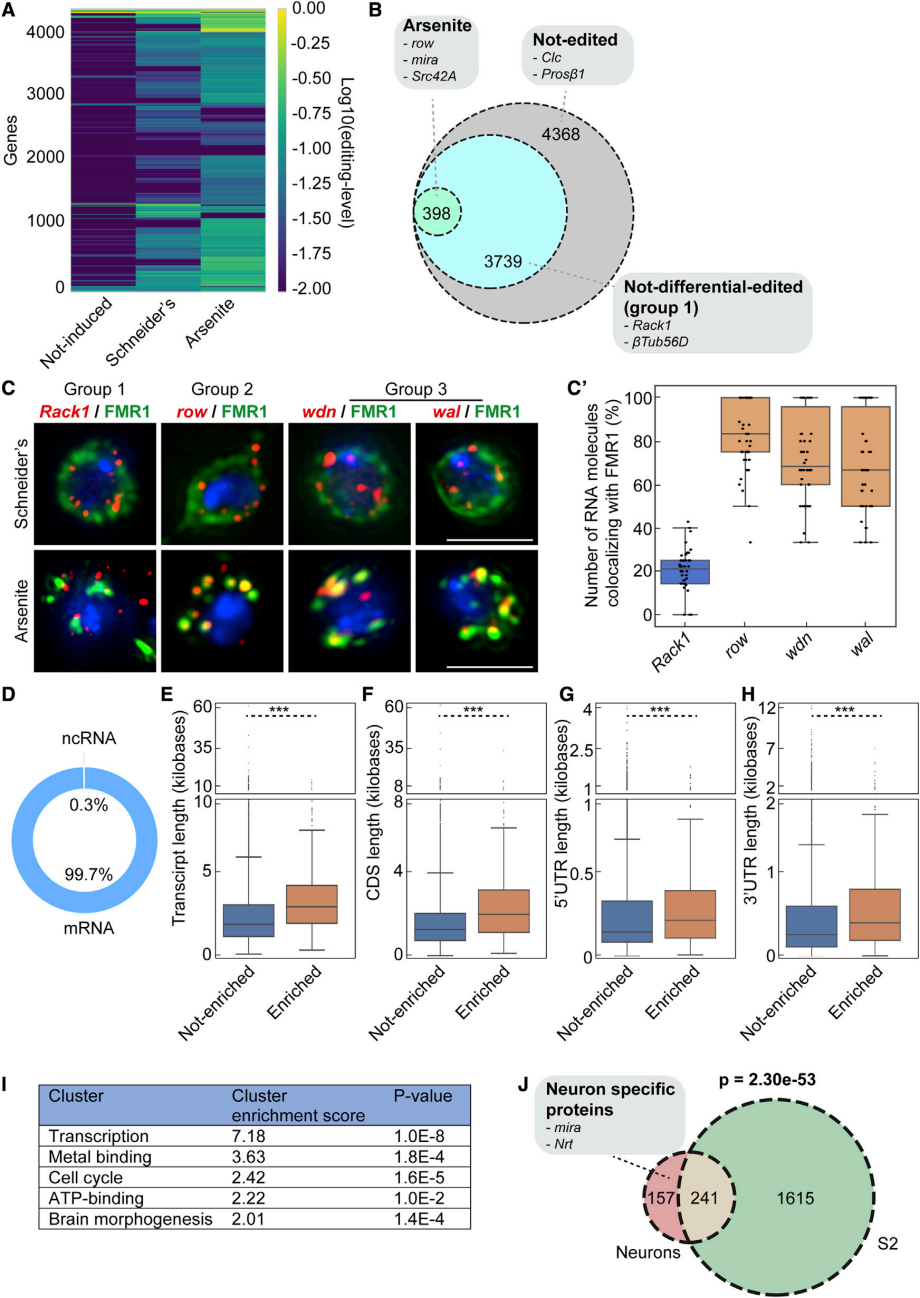
RNA editing through ADAR predicts the stress granule transcriptome of dissociated *Drosophila* neurons (A) Heatmap displaying the editing level per gene and per condition. (B) Venn diagram depicting the number of RNAs that were significantly (p < 0.01) more edited upon arsenite after applying the Empirical Bayes statistical test on the data set. (C) Visualization of *row*, *wdn*, *wal*, and *Rack1* mRNA (red) by smFISH in wild-type neurons. *Row*, *wdn*, and *wal* were all more significantly (p < 0.01) more edited upon arsenite compared with Schneider’s in neurons, while *Rack1* is not. Stress granules are marked with endogenous FMR1 (green). Quantification of the number of RNAs co-localizing with FMR1 (stress granules) in (C’). (D) Chart depicting the types of RNAs in stress granules. (E–H) Boxplots displaying the length of transcripts (E), CDS (F), 5′UTRs (G), and 3′UTRs (H) of the mRNAs predicted to be in stress granules (recruited) and of the mRNAs that were predicted not recruited in neurons. ∗∗∗p value < 0.001 (Mann-Whitney *U* test). (I) Gene enrichment analysis of the RNAs predicted to be localized in stress granules upon arsenite in neurons (cluster analysis using DAVID). (J) Venn diagram depicting the overlap of RNAs significantly (p < 0.01) more edited upon arsenite in S2 cells and in *Drosophila* neurons. Scale bar: 3 μm (C).

We sequenced the total RNA isolated from biological triplicates of dissociated neurons from the uninduced, Schneider’s, and arsenite-stressed conditions. Across all conditions, we detected the editing of 4,137 RNAs including 870 endogenous edited RNAs at very low levels (Figure 7A and Table S3). Of the 4,137 edited RNAs, 3,739 RNAs, such as *Rack1*, were not differentially edited (group 1) and are predicted not to be in stress granules (Figure 7B and Table S3). Conversely, 398 RNAs were significantly more edited upon arsenite stress (p < 0.01, Empirical Bayes test) and are predicted to be in stress granules (Figures 7B and Table S3).

As for S2 cells, group 2 RNAs (edited in basal conditions but significantly more upon arsenite), contain 326 (82%) RNAs, such as *row*, *mira*, and *Src42A*. Group 3 (only edited upon arsenite) contains 72 RNAs (18%), such as *wal* and *eIF6*.

As for S2 cells, RNAs from both group 2 and 3 are predicted to be in stress granules. To validate this, we used smFISH as above, and show that *row*, *wdn*, and *wal* RNA are localized in arsenite-induced stress granules in the neurons of wild-type flies (Figures 7C and 7C’). Conversely, and similar to S2 cells, *Rack1* is not enriched in stress granules, as predicted. These data indicate that, as for S2 cells, transcripts that were predicted to be in stress granules through RNA editing are indeed localized in stress granules.

### Arsenite-induced stress granules in S2 cells and neurons share the same RNAs

The majority (∼99.7%) of RNAs predicted to be recruited to neuronal stress granules are coding mRNAs with only ∼0.3% of ncRNAs (Figure 7D). They are on average longer than mRNA not recruited in stress granules (Figures 7F–7H). The gene analysis revealed that, as in S2 cells, the predicted stress granule RNAs show an enrichment for genes encoding ATP binding, transcription, cell cycle factors (Figure 7I).

Performing a parallel analysis in *Drosophila* S2 cells and neurons, we established that 61% of the 398 RNAs predicted in neuronal stress granules are also predicted to be in stress granules in S2 cells (Figure 7J). GO-term analysis confirmed an enrichment for neuron-related factors/proteins, such as *mira* and *Nrt* in the remaining 39%.

## Discussion

Here, using our adaptation of RNA editing via hyperTRIBE (using FMR1-ADARcd) combined to VASA-seq (a sequencing method allowing the detection of editing events along the whole RNA sequence) (Hollfelder et al., 2022), and smFISH, we show that the RNA content of stress granules can be reliably identified not only in bulk cells but also in single *Drosophila* S2 cells, and in tissues *(Drosophila* neurons).

### Identification of stress granule transcriptome in bulk S2 cells

In bulk S2 cells, we identified 1,856 potential stress granule RNAs, seven of them validated by smFISH. Importantly, only 25% of these RNAs are expected to be clients of FMR1. As in mammalian (Khong et al., 2017; Namkoong et al., 2018; Padrón et al., 2019) and yeast cells (Khong et al., 2017), the majority of the stress granule RNAs in S2 cells are mRNAs that are longer (here 30%) than their non-recruited counterparts. Longer mRNAs may potentially have more binding sites for RBPs that are proposed to be major driving factors in stress granule partitioning (Maharana et al., 2018; Matheny et al., 2021; Banani et al., 2017; Jain and Vale, 2017; Molliex et al., 2015; Murray et al., 2017; Patel et al., 2015). They may also extend beyond the stress granule physical boundaries where they might interact with, and pull, cytoplasmic RNAs within stress granules (Moon and Parker, 2018). Indeed, RNA-RNA interactions are proposed to contribute to stress granule assembly (Van Treeck and Parker, 2018), and this is reinforced by the notion that RNAs themselves can phase separate (Langdon et al., 2018).

Conversely, we found fewer ncRNAs (0.4%) in the *Drosophila* S2 stress granule transcriptome when compared with what is reported for mammalian stress granules (22%) (Khong et al., 2017). This may partially be explained by the fact that *Drosophila* cells contain a number of ncRNA loci 8-fold lower than mammalian cells (1,119 and 9,277, respectively) (Derrien et al., 2012; Young et al., 2012). Last, In agreement in mammalian cells (Khong et al., 2017), pre-mRNAs are not predicted to be in stress granules.

GO analysis of the S2 cell stress granule mRNAs shows enrichment in ATP binding, transcription, cell cycle, and RNA splicing factors that also compare well the stress granule mRNA complement in NIH3T3 cells (Namkoong et al., 2018). These similarities indicate that a common core of stress granule RNAs might be conserved across evolution.

### Stress granule transcriptome in single S2 cells

When compared with other published techniques, the RNA-editing-based method we used here has the major advantage to require neither stress granule isolation, nor the RNA immunoprecipitation. We found that the edited RNAs overlap by 64% between the pseudobulk (360 cells) and the bulk (2 million cells), showing the reliability and the potential of the approach. The analysis of these editing events did not reveal a heterogeneity between cells, either those kept in basal conditions, or those exposed to arsenite. Perhaps sequencing deeper would reveal a heterogeneity within a condition. However, in cultured S2 cells, this is not necessarily expected. Our method could therefore be used for the detection of stress granule RNAs in rare cells *in vivo*, either forming a small tissue or disseminated into larger tissues.

### *Drosophila* neurons

In this regard, using genetic tools to control spatial and temporal expression of ADARcd, we show that hyperTRIBE can be adapted to specifically identify the arsenite-triggered stress granule transcriptome in *Drosophila* neurons. Neuronal stress granules contain fewer RNAs (∼5% of the expressed transcriptome) than S2 cells (∼25%). Ruling out technical differences in ribosome removal and sequencing, these differences might be due to a lower editing efficiency per transcript in neurons. Indeed, there were fewer editing events per transcript in neurons when compared with S2 cells, although the number of edited transcripts was roughly the same in both, as was the expression of FMR1-ADARcd (estimated by immunofluorescence). The reasons for this lower editing efficiency are not clearly understood.

Despite these differences, 398 RNAs predicted to be in neuronal stress granules have been identified and three were validated (*row*, *wdn*, and *wal*). As in S2 cell stress granules, they are mainly longer mRNAs displaying a similar enrichment for ATP binding, transcription, and cell cycle factors.

The identification of the stress granule transcriptome could be extended to mammalian cells and tissues by using TET-on inducible systems or by specific conditional CRE promotors to ensure tissue and time specificity. Recently, hyperTRIBE was shown to be able to detect protein-RNA interactions in mammalian cells (Biswas et al., 2020). This could also be easily followed by single-cell analysis, to not only investigate the stress granule RNA content in specific cell type, but also to address their heterogeneity of their response.

### Single-molecule FISH

Of the potential stress granule RNAs that we have identified, nine have been validated by smFISH in S2 cells and neurons, plus two, which are predicted not to be. An extended validation could be performed using multiplex smFISH (Maynard et al., 2020), potentially revealing whether stress granules are populated with all RNAs identified, or a subset of them, and in which ratio. Our smFISH in S2 cells has begun to reveal such a stress granule heterogeneity (Figures S5A and S5B). If this was confirmed in a systematic manner, understanding what governs this heterogeneity could reveal important principles governing stress granule formation.

### Why are RNAs recruited to stress granules?

One of the large questions in the stress granule field is why mRNAs are recruited in stress granules. Besides their structural role that has been proposed recently (Van Treeck and Parker, 2018; Langdon et al., 2018), one general hypothesis is that stress granules form upon stress to protect RNAs from degradation after translation stalls and that RNAs are no longer covered in ribosomes. When stress is relieved, these protected and capped mRNAs recruited to stress granules could be immediately translated, enabling the cells to thrive again and gain a fitness advantage. In this regard, stress granule formation has been shown to allow cell survival upon stress and thriving upon stress relief (Jevtov et al., 2015; Riback et al., 2017; Kroschwald and Alberti, 2017). The enrichment in RNAs encoding ATP binding, cell cycle, and transcription factors may be consistent with this notion.

However, translation initiation of the RNAs stored in stress granules has been shown not to be required for growth, as translation can resume before stress granule clearance is complete (Loschi et al., 2009; Walters et al., 2015). Furthermore, a recent study suggests that the mRNAs that are upregulated upon oxidative stress are vastly excluded from stress granules (Singh et al., 2021), strengthening the notion that stress granules might not be needed to protect RNA from degradation, but simply store them. Last, contrary to the finding that stress granules do not contain fully assembled ribosomes (only 40S) (Kedersha et al., 1999; Anderson and Kedersha, 2002), single-molecule imaging has revealed that the translation machinery can be activated in stress granules (Mateju et al., 2020). This suggests that although general translation is inhibited, stress granules could be a hub for translating specific RNAs, perhaps related to coping with stress.

As a proof of principle, using HyperTRIBE opens avenues to refine the specific core of common stress granule RNAs upon different stress and understand their role and their features. It is the first method to identify stress granule RNAs in single cells and in tissues, and could be particularly important to reveal heterogeneity between cells, within primary cells or tissues in their response to different stress. Importantly, this method could also allow the identification the RNA content of other RNA-based phase-separated assemblies, whether formed upon stress or existing in basal conditions, such as neuronal granules (Formicola et al., 2019; Kiebler and Bassell, 2006), posterior granules in *Drosophila* oocytes (Trcek and Lehmann, 2019; Bose et al., 2021) and P granules in *Caenorhabditis elegans* (Aoki et al., 2021; Lee et al., 2020), whose RNA content has so far been difficult to isolate and study.

### Limitations of the study

However successful, this proof of principle presents several limitations. First, cells and tissues need to express ADARcd through transfection or generation of transgenic animals. Second, one of the requirements is to limit the basal editing (in the absence of stress even if RNA do not have a long half-life) by controlling the timing and temporal (*in vivo*) expression of ADARcd. In *Drosophila* cells and tissues, we used the metallothionein-inducible promoter and the geneswitch system, respectively. Photoactivatable (light oxygen voltage) FRM1-ADARcd would also be envisioned for specific activation in stress granules, thus, to restrict even more the background (Wu et al., 2009). Third, the calculation editing frequencies could be done differently. Here, we calculated the average editing frequencies for a gene by extracting the percentage of mutated bases at each identified variant position, for each sample, and averaged the numbers (see method details section, Figure S4D). However, the editing frequency could be calculated by extracting the number of all edits over all variant positions for one gene and dividing this by the sum of the total positions (see STAR Methods for further discussion of this, with examples). This strategy could in particular be important for genes with considerably varying read depths at edited positions across a gene. In this study, using this alternative strategy gave slightly different results but did not affect the number and the types of RNAs predicted to be localized in stress granules (99.99% overlap). Fourth, certain RNAs may be covered with RBPs, therefore blocking the editing sites. Fifth, even though our validation by smFISH indicates that the RNAs predicted to be in stress granules are localized to these coalescences, it is possible that due to the translational arrest during arsenite treatment, these RNAs are edited in the cytosol by FMR1-ADARcd-V5 before they coalesce into stress granules. To assess how many and which RNAs are edited by ADARcd in the cytoplasm in arsenite irrespective of their coalescence into stress granules, NES-ADAR (instead of FMR1-ADARcd) could be expressed. Sixth, once edited, a given RNA molecule might leave the stress granule and return to the cytoplasm or to P-bodies (Zhang et al., 2011; Mollet et al., 2008), leading to the identification of RNAs that are only transiently residing in stress granules. However, the predicted stress granule RNAs we have tested by smFISH are enriched in stress granules, not only present, suggesting that they are true resident. Last, ADARcd was only tagged to one RBP (FMR1), thus potentially biasing our analysis toward FMR1 clients. Even if it is not the case, using another stress granule protein that is not an RBP would further validate our findings.

## STAR★Methods

### Key resources table

**Table:** 

REAGENT or RESOURCE	SOURCE	IDENTIFIER
**Antibodies**		

Mouse anti-dFMR1	DSHB	Cat#5A11
Mouse anti-caprin	(Reich and Papoulas, 2012)	Ophelia Papoulas lab
Mouse anti-V5	ThermoFisher	Cat#R960-25; RRID: AB_2556564
Rabbit anti-V5	Sigma-Aldrich	Cat#V8137; RRID: AB_261889
Rat anti-elav	DSHB	Cat#7E8A10; RRID: AB_528218
Mouse anti-repo	DSHB	Cat#8D12; RRID: AB_528448
Goat anti-mouse Alexa 488	Invitrogen	Cat#A11001; RRID: AB_2534069
Donkey anti-rabbit Alexa 568	Invitrogen	Cat#A10042; RRID: AB_2534017
Donkey anti-mouse Alexa 568	Invitrogen	Cat#A10037; RRID: AB_2534013
Donkey anti-mouse Alexa 647	Invitrogen	Cat#A31571; RRID: AB_162542
Goat anti-rat Alexa 633	Invitrogen	Cat#A21094; RRID: AB_2535749
Donkey anti-rabbit Alexa 647	Invitrogen	Cat#A31573; RRID: AB_2536183
Mouse anti-alpha-tubulin	Sigma-Aldrich	Cat#T5168; RRID: AB_477579
Sheep anti-mouse HRP conjugated	GE Healthcare	Cat#GENA931; RRID: AB_772210

**Chemicals, peptides, and recombinant proteins**		

Schneider’s Insect media	Sigma-Aldrich	Cat#S0146
Fetal bovine serum (suitable for insect)	Sigma-Aldrich	Cat#F4135
Sodium (meta)arsenite	Sigma-Aldrich	Cat#S7400
Hygromycin B	ThermoFisher	Cat#10687010
Copper(II) sulfate pentahydrate	Sigma-Aldrich	Cat#C7631
In-Fusion® Snap Assembly Master Mix	TaKaRa	Cat#638947
RU486	Sigma-Aldrich	Cat#M8046
Poly-L-lysin	Sigma-Aldrich	Cat#P1274
Collagenase type I	Sigma-Aldrich	Cat#SCR103
Papain from papaya latex	Sigma-Aldrich	Cat#P4762
Gelatin from cold water fish skin	Sigma-Aldrich	Cat#G7765
Dextran sulfate sodium salt from Leuconostoc spp. Mw >500,000	Sigma-Aldrich	Cat#D8906
Atto565-NHS	Atto-tec	Cat#AD565
Amino-11-ddUTP	Lumiprobe	Cat#15040
TdT enzyme	ThermoFisher	Cat#EP0151
Linear acrylamide	ThermoFisher	Cat#AM9520

**Critical commercial assays**		

Effectene Transfection Reagent	Qiagen	Cat#301425
Pierce BCA protein assay kit	ThermoFisher	Cat#23225
Direct-zol RNA microprep kit	Zymo Research	Cat# R2063
Qubit RNA High Sensitivity assay kit	Thermofisher	Cat#Q32852
RNA 6000 Pico chip	Agilent Technologies	Cat#5067

**Deposited data**		

Raw and processed sequencing files	This study	GSE: GSE175782
Drosophila reference genome (BDGP6)	BDGP6	https://www.ensembl.org/Drosophila_melanogaster/Info/Index
Raw data processing pipeline for TRIBE	This study	https://doi.org/10.5281/zenodo.6419119

**Experimental models: Cell lines**		

*D*. *melanogaster*: Cell line S2	Catherine Rabouille lab	
*D*. *melanogaster*: Cell line S2 pMT-FMR1-ADARcd[E488Q]-V5	This study	N/A

**Experimental models: Organisms/strains**		

*D*. *melanogaster*: strain w[1118]	Catherine Rabouille lab	N/A
*D*. *melanogaster*: y[1] w[∗]; P{w[+mC]=elav-Switch.O}GSG301	Bloomington Drosophila Stock Center	BDSC:43642
*D*. *melanogaster*: w[1118]; P{w[+mC]=UAS-FMR1-ADARcd[E488Q]-V5-2xT2A-EGFP/CyO	This study	N/A

**Oligonucleotides**		

Oligos for cloning, library prep and smFISH, see Table S4	Alexander van Oudenaarden lab	N/A
TMR-oligo(dT)30x	IDT	N/A

**Recombinant DNA**		

pMT-FMR1-V5	(Aguilera-Gomez et al., 2017)	Catherine Rabouille lab
pUASt	N/A	Catherine Rabouille lab
pMT-2xT2A	N/A	Alberto Baena Lopez lab
pEGFP	Addgene	Cat#13031
Gblock containing ADARcd E488Q	IDT	N/A

**Software and algorithms**		

tRNAscan-SE version 2.0.7	(Lowe and Chan, 2016)	http://lowelab.ucsc.edu/tRNAscan-SE/
cutadapt version 3.2	(Martin, 2011)	https://cutadapt.readthedocs.io/en/stable/
bwa aln version version 0.7.17-r1188	(Li and Durbin, 2009)	http://bio-bwa.sourceforge.net/
STARSolo version 2.7.7a	(Kaminow et al., 2021)	https://github.com/alexdobin/STAR/blob/master/docs/STARsolo.md
UMI-tools version 1.1.1	(Smith et al., 2017)	https://pypi.org/project/umi-tools/
GATK version 4.1.9.0	(DePristo et al., 2011)	https://gatk.broadinstitute.org/hc/en-us/sections/360010932391-4-1-9-0
Python 3.7.0	Python	https://www.python.org/downloads/release/python-370/
R-3.6.0	R	https://cran.r-project.org/bin/windows/base/old/3.6.0/
Imaris Image Analysis Software version 9.3.0	Bitplane	https://imaris.oxinst.com/versions/9-3
Fiji	ImageJ/NIH	https://imagej.net/Fiji
DeconvolutionLab2	(Sage et al., 2017)	http://bigwww.epfl.ch/deconvolution/deconvolutionlab2/

### Resource availability

#### Lead contact

Further information and requests for resources should be directed to and will be fulfilled by the lead contact, Catherine Rabouille (c.rabouille@hubrecht.eu).

#### Materials availability

pMT-FMR1-ADARcd-V5 and pUAS-FMR1-ADARcd-V5-T2A-EGFP plasmids will be provided upon request.

### Experimental model and subject details

Wild type *Drosophila* S2 cells were cultured in Schneider’s medium (Sigma Aldrich) supplemented with 10% insect tested fetal bovine serum (Sigma Aldrich) at 26°C. This medium is referred to as Schneider’s. *Drosophila* S2 cells stably transfected with pMT-FMR1-ADARcd-V5 (see below) were grown in Schneider’s supplemented with 300 μg/mL hygromycin B (ThermoFisher).

Flies were reared on rich fly food (https://bdsc.indiana.edu/information/recipes/germanfood.html) and maintained at 25°C with 70% humidity with a 12 h light/dark cycle. Brains were isolated from both male and female larvae (no sexual selection).

### Method details

#### Arsenite treatment

Wild type *Drosophila* S2 cells or *Drosophila* S2 cells stably transfected with pMT-FMR1-ADARcd-V5 (see below) were plated to a concentration of 1.5 × 10^6^ cells per well in a 12-wells plate containing a coverslip and allowed to adhere for 1 h at 26°C in Schneider’s. The expression of FMR1-ADARcd-V5 was induced by addition of 1 mM CuSO_4_ for 4 h at 26°C. This was followed by either a further incubation in Schneider’s (basal condition) or incubation with 500 μM NaAsO_2_ (arsenite) for 4 h at 26°C.

#### Induction of geneswitch, dissociation *Drosophila* brain and arsenite treatment

To induce the expression of FMR1-ADARcd-V5-T2A-EGFP specifically in neurons, we crossed homozygous UAS-FMR1-ADARcd-V5-T2A-EGFP male flies (on second chromosome) with homozygous elav-geneswitchGal4 (43642, BDSC) female flies (on third chromosome). Eggs were laid in large tubes containing rich fly food. After two days, flies were removed and the tubes were further incubated for 3 days at 25°C. 30 wandering third instar UAS-FMR1-ADARcd-V5-T2A-EGFP; *elav*-GeneSwitch-Gal4 larvae were collected in a 2 mL Eppendorf tube, washed 2 times with MiliQ, washed once with 80% ethanol, and incubated in 3 mg/mL RU486 (in 80% ethanol, M8046, Sigma Aldrich) for 10 min to activate the geneswitch. Larvae were transferred back to a fresh apple juice plate and allowed to recover for 14 h in the dark at RT (20°C). At least 20 third instar UAS-FMR1-ADARcd-V5-T2A-EGFP; *elav*-GeneSwitch-Gal4 larvae were collected, washed once with PBS, and dissected. Their brains were isolated in ice cold Schneider’s media in the lid of 6 cm dish and transferred to an Eppendorf tube containing ice cold Schneider’s. To dissociate the brains, the isolated brains were washed 2 times with Rinaldini solution (Rinaldini, 1959), and incubated with 1 mg/mL collagenase I and papain (Sigma Aldrich) in Rinaldini solution for 1 h at 30°C in a thermomixer. Schneider’s was then added and brains were washed 2 times with Schneider’s on ice. Brains were disrupted manually by pipetting up and down 75 times with a 200 μL pipet tip. The tissue pieces were forced through a cell-strainer FACS tube (BD Falcon) and either plated on poly-L-lysin (P1274, Sigma Aldrich) coated coverslips for immunofluorescence (IF) and single-molecule FISH (smFISH), or in a well of a 12 well plates for RNA isolation (see below). Upon adhesion for 1 h at RT in the dark (20°C), the neurons were either incubated in Schneider’s or stressed with 500 μM NaAsO_2_ for 4 h at 26°C.

#### RNA isolation from S2 cells and dissociated brain cells

*Drosophila* S2 cells stably transfected with pMT-FMR1-ADARcd-V5 were plated to a concentration of 3 × 10^6^ cells per well in a 6-wells plate 16 h before the experiment. The expression of FMR1-ADARcd-V5 was induced for 4 h followed by arsenite treatment as described above. The dissociated brain cells came from 20 third instar larval brains and were treated as above. The media was removed and Trizol (Zymo Research) was added to lyse cells. RNA was extracted from the Trizol using the Direct-zol RNA microprep kit (Zymo Research) following manufacturer’s instructions. The RNA concentration was determined using the Qubit RNA High Sensitivity assay kit (Thermofisher). The quality of the RNA was analyzed by running the samples on an RNA 6000 Pico chip (Agilent Technologies) on the Bioanalyzer (Agilent Technologies).

#### FACS – Single-cell sorting

S2 cells were harvested without the requirement of dissociation (S2 cells grow as single cells). Single-S2-cell suspensions were resuspended in PBS0 with 1 μg/mL DAPI, and passed through a 20-μm mesh. A total of 360 single cells were index sorted using a BD FACS Influx in 384-well hardshell plates (BioRad) that were pre-filled with 5 μL of light mineral oil (Sigma Aldrich) and 50 nL of 0.25 μM CelSeq2 primer (CS2001 to CS2384, Table S4) (Muraro et al., 2016). Doublets, debris, and dead cells were excluded by gating forward and side scatter in combination with the DAPI channel.

#### Library preparation for VASA-seq

Library preparation was performed using VASAseq (Hollfelder et al., 2022), a high-sensitivity, strand-specific method for full-length RNA-seq. Briefly, RNA goes through cDNA synthesis and is linearly amplified using *in vitro* transcription. Amplified RNA (aRNA) is then depleted of ribosomal RNA sequences, and converted to libraries for sequencing.

For cDNA synthesis on bulk RNA, 6 ng of total RNA was mixed with 9 μL fragmentation buffer [1.7× of 5× First Strand Buffer (Invitrogen), 125 nM cDNA synthesis primer mix (CS2_001 to CS2_012; Integrated DNA Technologies; Table S2), 0.1% Triton-X 100, 1:125000 ERCC Spike-In Mix (ThermoFisher)], and was incubated at 25°C for 60 min 55°C for 10 min, cooled to 4°C, then snap heated to 85°C for 3 min and snap chilled on ice. Next, 5 μL of end-repair and A-tailing brew [0.6× of 5× First Strand Buffer, 20 mM DTT (Invitrogen), 7.5 μM ATP, 0.075 U/μL *E*. *coli* Poly(A) polymerase (New England Biolabs), and 1 U/μL T4 Polynucleotide Kinase (New England Biolabs)] was added to the fragmented RNA and incubated at 37°C for 60 min and held at 4°C. End-repaired and A-tailed RNA was then reverse transcribed by adding 5 μL of reverse transcription mix [2 mM dNTPs (Promega) and 16 U/μL Super-Script III (Invitrogen)] and incubating at 50°C for 60 min and holding at 4°C. Second-strand synthesis was next performed by adding 110 μL of second-strand brew [1.14× of 5× Second-Strand Buffer (Invitrogen), 0.23 mM dNTPs, 0.32 U/μL *E*. *coli* DNA Polymerase I (Invitrogen), and 0.018 U/μL RNase H (Invitrogen)] and incubating at 16°C for 2 h, 85°C for 20 min, and holding at 4°C. Double-stranded cDNA was cleaned up using 8× diluted AMPure XP beads (Beckman) at a 1:1 ratio and resuspended in 6.4 μL of water.

When processing single cells, the same brews and incubation procedures for the cDNA synthesis steps were used, however volumes were scaled down and dispensed with the Nanodrop II (Innovadyne Technologies Inc.). Frozen 384-well plates were thawed on ice, and 50 nL of fragmentation buffer, end-repair and A-tailing brew, and reverse transcription mix were each sequentially added, followed by 550 nL of second-strand brew. Plates were spun at 2000 ×g after each liquid transfer step. Plates were pooled, and double-stranded cDNA was cleaned up using 8× diluted AMPure XP beads (Beckman) at a 1:1 ratio and resuspended in 6.4 μL of water.

The purified cDNA was then *in vitro* transcribed in a 16 μL reaction at 37°C for 14 h using the MegaScript T7 *in vitro* transcription kit (Invitrogen) following the manufacturer’s directions. This reaction was stopped by adding 6 μL ExoSAP-IT (Invitrogen) and incubating at 37°C for 15 min. Amplified RNA was purified using AMPure XP beads at a 1.8:1 ratio and resuspended in 10 μL water.

Amplified RNA was then depleted of ribosomal sequences by adding 600 ng of purified aRNA to hybridization brew [250 mM Tris HCl pH 7.5, 500 mM NaCl, 12.5 μM ribosomal RNA depletion probes (Integrated DNA Technologies; dmelrRNA_; Table S2)] in a 10 μL reaction, incubating at 95°C for 2 min, decreasing the temperature to 45°C at a rate of 0.1°C/s, and holding at 45°C. Ten microliters of thermostable RNase H mix [100 mM Tris HCl pH 7.5, 200 mM NaCl, 40 mM MgCl_2_, 1 U/μL Hybridase Thermostable RNase H (Epicenter)] was then pre-warmed to 45°C and added to the probe-annealed samples and incubated at 45°C for 30 min and cooled to 4°C. Ribosomal probes were then removed by adding 30 μL of DNase mix [1.66 mM CaCl_2_, 0.133 U/μL RQ1 DNase (Promega)] and incubated at 37°C for 30 min. The resulting rRNA-depleted aRNA was cleaned up with AMPure XP beads at a 1.6:1 ratio and resuspended in 6 μL of water.

Finally, ribosomal-depleted aRNA was converted to sequencing libraries. First, 1 μL of RA3 adapter (Table S2) was added to the purified rRNA-depleted aRNA, denatured at 70°C for 2 min, and snap chilled on ice. Next, 4 μL of ligation brew [2.5× of 10× T4 RNA Ligase Reaction Buffer (New England Biolabs), 50 U/μL T4 RNA Ligase 2 Truncated (New England Biolabs), and 10 U/μL RNaseOUT (Invitrogen)] was added and incubated at 25°C for 1 h and held at 4°C. Adapter-ligated product was reverse transcribed by adding 3 μL of primer-dNTP mix [3.33 μM dNTPs and 13.33 μM RTP primer (Table S2)], denaturing at 65°C for 5 min and snap-chilling on ice, followed by adding 8 μL of reverse-transcription mix [2.5× of 5× First Strand Buffer, 12.5 mM DTT, 5 U/μL RNaseOUT, 25 U/μL Super-Script III], incubating at 50°C for 1 h, 70°C for 15 min, and holding at 4°C. RNA was next degraded by adding 1 μL RNase A (ThermoFisher), incubating at 37°C for 30 min and holding at 4. Complementary DNA was cleaned up using AMPureXP beads at a 1:1 ratio and eluted in 20 μL water. Finally, libraries were amplified by adding 40 μL of PCR brew [1.25 × of 2× NEBNext High Fidelity Reaction Mix (New England Biolabs), 0.5 μM RP1, and 0.5 μM Library Index RPI (RPI01-48, Table S2)] to half of the purified cDNA, and incubated at 98°C for 30 s, followed by seven to nine cycles of of 98°C for 10 s, 60°C 30 s, 72°C for 30 s, and then a final incubation at 72°C for 10 min and holding at 4°C. PCR-amplified libraries were cleaned up twice with AMPure XP beads, both with a 0.8:1 ratio.

Quality of the final libraries was assessed using the Agilent High Sensitivity DNA bioanalyzer (Agilent) and quantified with the Qubit (Invitrogen) before sequencing.

#### Sequencing

Libraries were sequenced using v2.5 chemistry on a NextSeq 500 (Illumina).

#### Data analysis – Reference genomes and annotations

The Drosophila reference genome (BDGP6), annotations, and known variants were obtained from Ensembl release 95. Ribosomal RNA sequences used for read depletion were downloaded from FlyBase. The reference genomes were prepared for alignment by masking all tRNA genes and pseudogenes and including unique mature tRNAs genes as artificial chromosomes. tRNA genes and pseudogenes were identified using tRNAscan-SE (version 2.0.7) using the eukaryotic model (-HQ) and the vertebrate mitochondrial model (-M vert -Q).

#### Data analysis – Read processing

Adapter and homopolymer sequences were first trimmed from read 2 using cutadapt (version 3.2) with -U 6 -U -1 --times 5 -m 15 -A GTTCAGAGTTCTACA -A AAAAAAAAAAAAAAA -A TTTTTTTTTTTTTTT -A CCCCCCCCCCCCCCC -A GGGGGGGGGGGGGGG. Next, trimmed reads were depleted of rRNA sequences by aligning to a ribosomal reference using bwa aln (version 0.7.17-r1188) and bwa mem, and discarding any read that aligned using either method. Depleted reads were then two-pass aligned to the reference genome using STARSolo (version 2.7.7a) with a 125-nt overhang with the following parameters: --seedSearchStartLmax 10 --alignIntronMax 1000000 --outFilterType BySJout --alignSJoverhangMin 8 --outFilterScoreMin 0 --outFilterMultimapNmax 1 --chimScoreSeparation 10 --chimScoreMin 20 --chimSegmentMin 15 --outFilterMismatchNmax 5. Aligned reads were de-duplicated with UMI-tools (version 1.1.1) using --spliced-is-unique --per-gene --per-cell.

#### Data analysis – Workflow variant identification and calculation mutation frequency

The non-randomness of the edited sites leads us to the following data processing workflow, resulting in a gene-wise editing metric (Figure S4C). Sequencing reads were first trimmed, aligned, and de-duplicated. The resulting de-duplicated alignments were merged and enter the GATK RNA-seq short variant discovery best-practices workflow using GATK (version 4.1.9.0). Reads with N in the alignment cigar were split into multiple alignments (SplitNCigarReads). Next, there were two rounds of base quality score recalibration (BaseRecalibrator, ApplyBQSR, and AnalyzeCovariates) and variant calling (HaplotypeCaller). The first round of recalibration masked all known reference variants, and the second additionally masked all novel variants. The exonic variants from the second round of variant calling were used for further downstream analyses. The variant positions were further selected based on hard cut-offs for quality (QUAL >250) and mutation frequency (AF < 0.95). Manual inspection on several genes revealed that the resulting positions were indeed capturing the rare, moderately edited positions that we previously observed (Figure S4A; *vertical light grey bars highlight identified variants*). For each of these variant positions, we then extracted the total number of mutated bases for each sample and used these to calculate their editing frequency. The editing frequencies of all identified variant positions for a gene were then averaged; this is reported as the “average editing frequency”. For instance, with reference to Figure S4D, 17 of the 53 “A” at position 1 are ‘G’; 23 of 58 “A” at position 2 are “G”, giving an editing frequency of 0.32 and 0.39, respectively, leading to an average of 0.358. Alternatively, the editing frequency for each gene can be calculated by taking the sum of all edits over all positions and divide this by the sum of the total bases over all positions in one gene. In the example shown in Figure S4C, the editing frequency would be calculated as (17 + 23)/(53 + 58) = 0.36. Note that the frequencies are very slightly different, but it did not affect the number and the types of RNAs predicted to be localized in stress granules (1856 in the used strategy versus 1855 using this alternative strategy, with 99.99% overlap). As mentioned in the discussion, using this alternative analysis strategy might be important when many genes are detected with low or varying read depths at edited positions across a gene.

#### Immunofluorescence (IF)

After treatments, S2 cells and dissociated brain cells were fixed with 4% paraformaldehyde (Sigma Aldrich) in PBS (pH 7.4) for 20 min. Cells were stained following the same protocol as described in (Zhang et al., 2021).

#### Western blot of FMR1-ADARcd-V5

A total of 3 × 10^6^ S2 cells expressing pMT-FMR1-ADARcd-V5 for 4 h were plated in a 6-wells plate per condition. Cells were further processed for Western blot analysis described in (Zhang et al., 2021).

#### Single-molecule FISH (smFISH)

Plated Wildtype S2 cells were first treated with arsenite (see above), fixed and labeled for endogenous FMR1 as described above in the IF section. After incubation with the secondary antibody, cells were washed 3 times with PBS and cells were post-fixed in 4% paraformaldehyde in PBS (pH 7.4) for 10 min. Following a washing 3 times in PBS, cells were further incubated for 5 min in 10% formamide (Thermofisher) in DEPC-treated water. They were then incubated overnight on a droplet containing one fluorescent smFISH probe (125 nM in 1% dextransulfate (D8906, Sigma Aldrich), 10% formamide (Thermofisher) in DEPC-treated water at 37°C) in a moistened chamber to avoid drying. Cells were washed 2 times for 30 min with 10% formamide (Thermofisher) in DEPC-treated water and mounted with Prolong antifade media (+DAPI) (ThermoFisher) on a microscope slide. All smFISH probes (Table S4) were labeled with Atto565 following (Gaspar et al., 2017). The DNA oligos were purchased from IDT. The TMR-oligo(dT)30x was also purchased from IDT.

#### Microscopy and image analysis

Immunofluorescence images were acquired on a TSC SP8 confocal microscope (Leica GmbH) with a 63× lens. For smFISH, a widefield Leica MM-AF microscope was used with a 100× lens. smFISH images were deconvoluted using the Regularized Inverse Filter algorithm with the DeconvolutionLab2 plugin (Sage et al., 2017) in ImageJ. The smFISH spots (each corresponding to a single RNA molecule, (Raj et al., 2008)) were counted using the spot count tool in the Imaris Image Analysis Software (Bitplane), and the percentage of spots in stress granules was estimated by their overlap in fluorescent signal with FMR1. At least, 20 randomly selected cells (N) were quantified per smFISH probe. The minimum and maximum display values were adjusted for each channel for visualization purposes.

For calculating the fraction of FMR1-ADARcd-V5 in stress granules, we used FIJI’s ‘’plot profile’’ plugin. The ratio of fluorescence intensity of FMR1 in stress granules and cytoplasm was calculated for 10 cells.

#### S2 cell death in arsenite stress and upon relief

A total of 2 × 10^6^ S2 cells per well in a 6-wells were plated and allowed to adhere for 1 h at 26°C in Schneider’s. Cells were treated with either Schneider’s or with arsenite for 4 h at 26°C in duplicate. Directly after the treatment, dead cells were stained with trypan blue and living cells were counted using a cell counter. After 4 h of arsenite, the growing medium was changed for Schneider’s, and living cells were counted at several time points after stress relief every 24 h up to 168 h.

In parallel, stress granule formation and dissolution were monitored by immunofluorescence (using FMR1 as marker). The number of cells displaying stress granules was scored again the total number of living cells in each time point.

### Quantification and statistical analysis

To identify the RNAs more edited upon stress conditions, we set that the editing frequency of RNAs from the “arsenite” triplicates should be significantly higher (p < 0.01 using the Empirical Bayes test (ebbr package in R)) than the editing frequency of same RNAs from the “basal triplicate”. The correlation (R2) between samples was calculated using the function pandas.dataframe.corr() in Python. A Mann-Whitney test (scipy.stats.mannwhitneyu) was used in Python to determine the significance between transcript length. To calculate the overlap between two datasets, we applied a hypergeometric test using the phyper function in R.

## Data Availability

• Raw RNA-seq data have been deposited and is available in the NCBI Gene Expression Omnibus under the accession number GSE175782. • All original code to process raw data have been deposited at GitHub (https://github.com/mvanins/stress_granule_RNA_manuscript) and Zenodo (https://doi.org/10.5281/zenodo.6419119). • Any additional information required to reanalyze the data reported in this paper is available from the lead contact upon request.

## Supplemental information

Document S1. Figures S1–S5

Table S1. RNA editing S2 cells, related to Figure 2

Table S2. Overlap with established FMR1 clients (Xu et al., 2018), related to Figure 2

Table S3. RNA editing neurons, related to Figure 7

Table S4. Oligos used for cloning, library preparation or smFISH, related to STAR Methods

Document S2. Article plus supplemental information
