# Supplementary material for: Identification of the stress granule transcriptome via RNA-editing in single cells and in vivo
Source: Cell Rep Methods. 2022 Jun 20;2(6):100235. doi: 10.1016/j.crmeth.2022.100235 (PMC9243631; doi:10.1016/j.crmeth.2022.100235)
Supplement: Document S1. Figures S1–S5 [file mmc1.pdf]

Cell Reports Methods, Volume *2*

Supplemental information

Identification of the stress granule transcriptome via RNA-editing in single cells and *in vivo*

Wessel van Leeuwen, Michael VanInsberghe, Nico Battich, Fredrik Salmén, Alexander van Oudenaarden, and Catherine Rabouille

Supplemental Figure S1: van Leeuwen et al, 2022

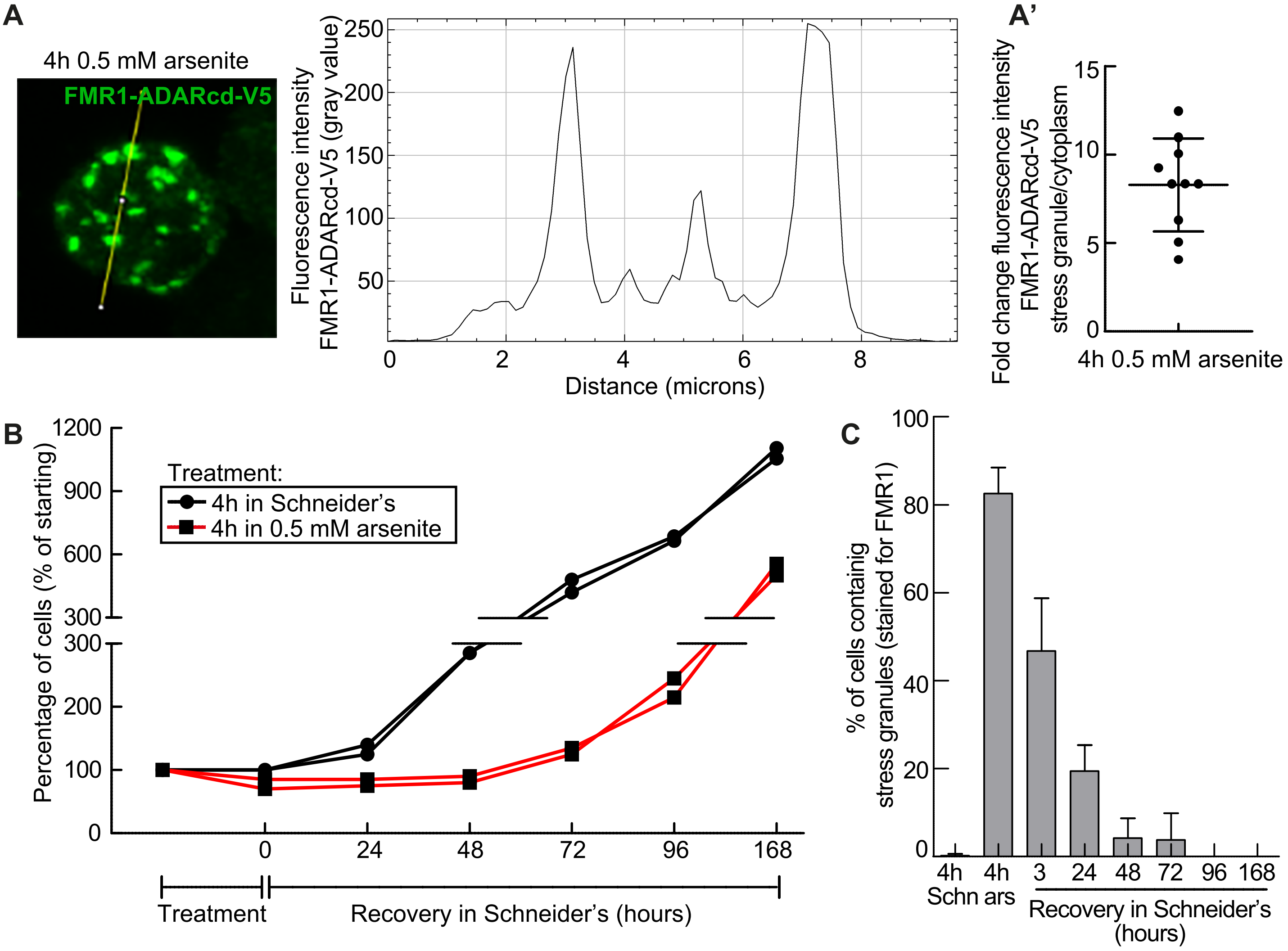

Supplemental Figure S2: van Leeuwen et al, 2022

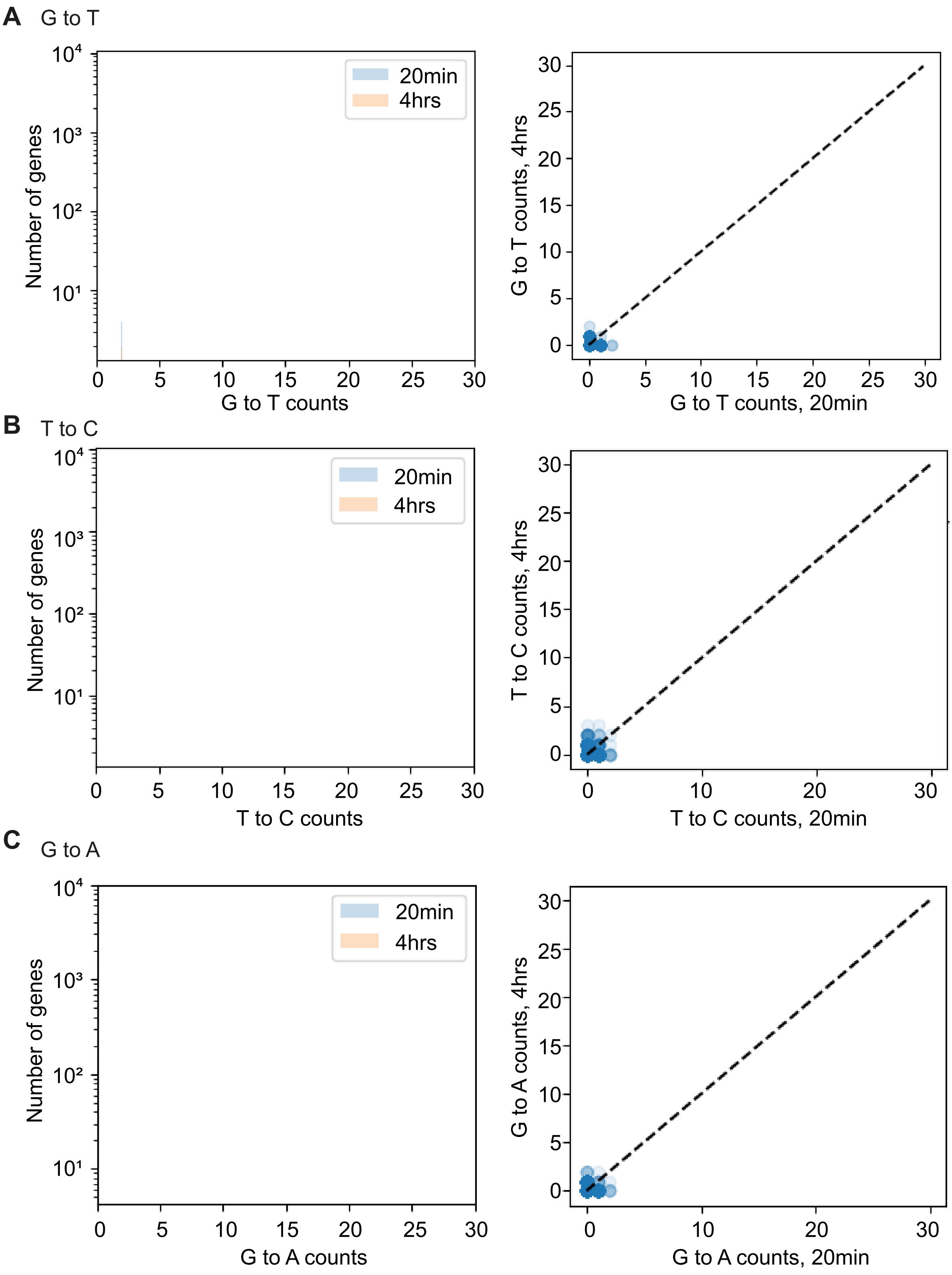

Supplemental Figure S3: van Leeuwen et al, 2022

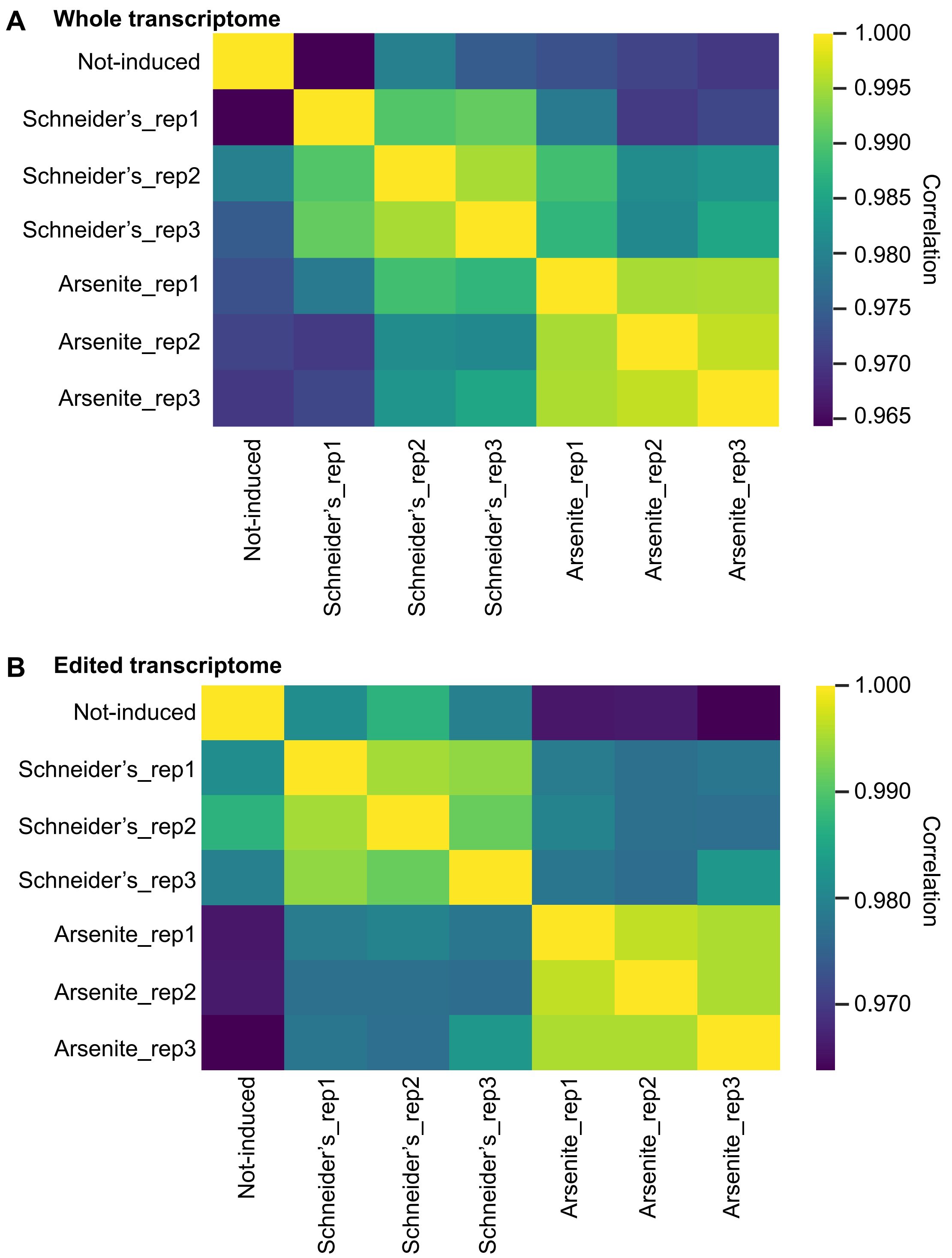

Supplemental Figure S4: van Leeuwen et al, 2022

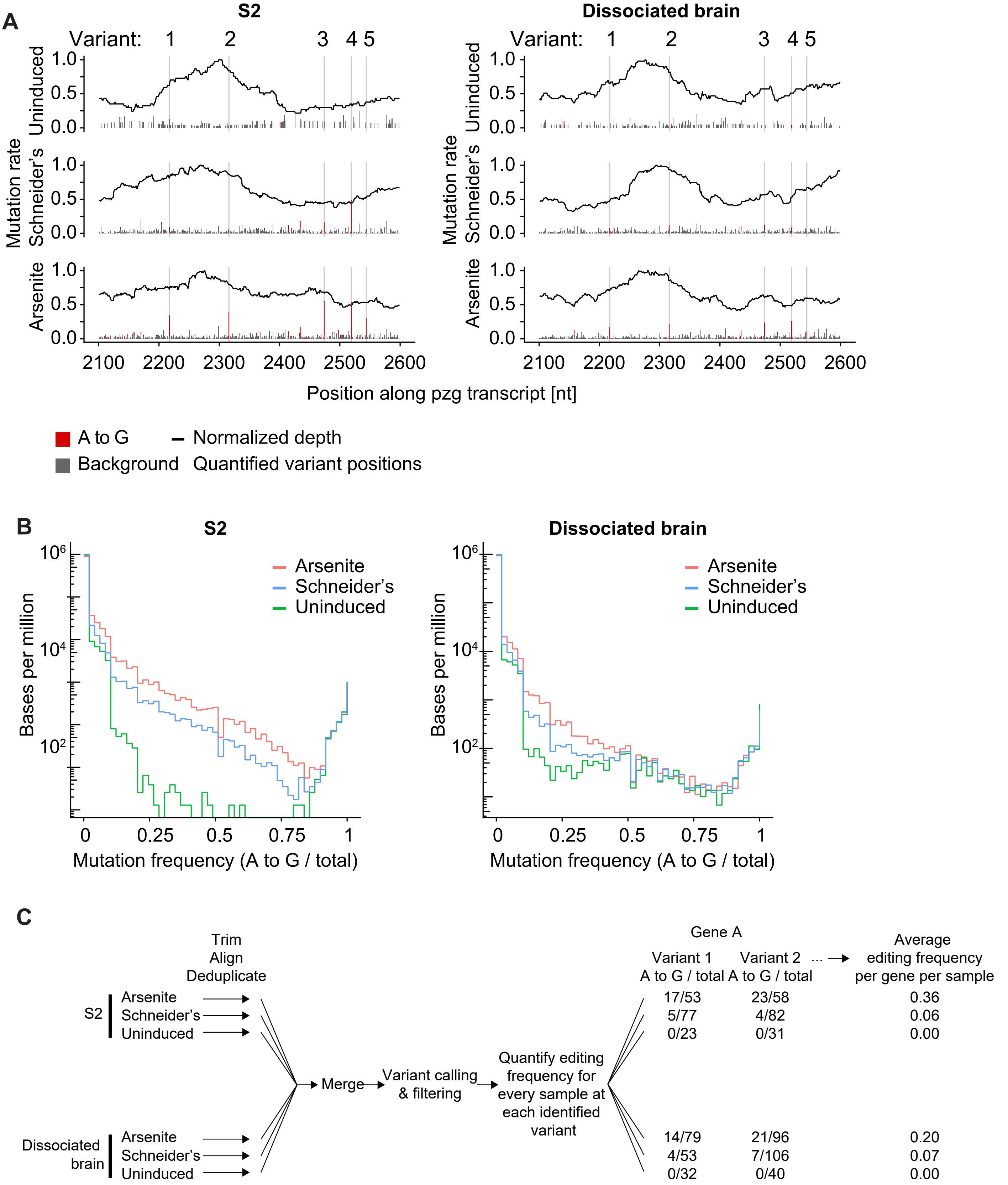

Supplemental Figure S5: van Leeuwen et al, 2022

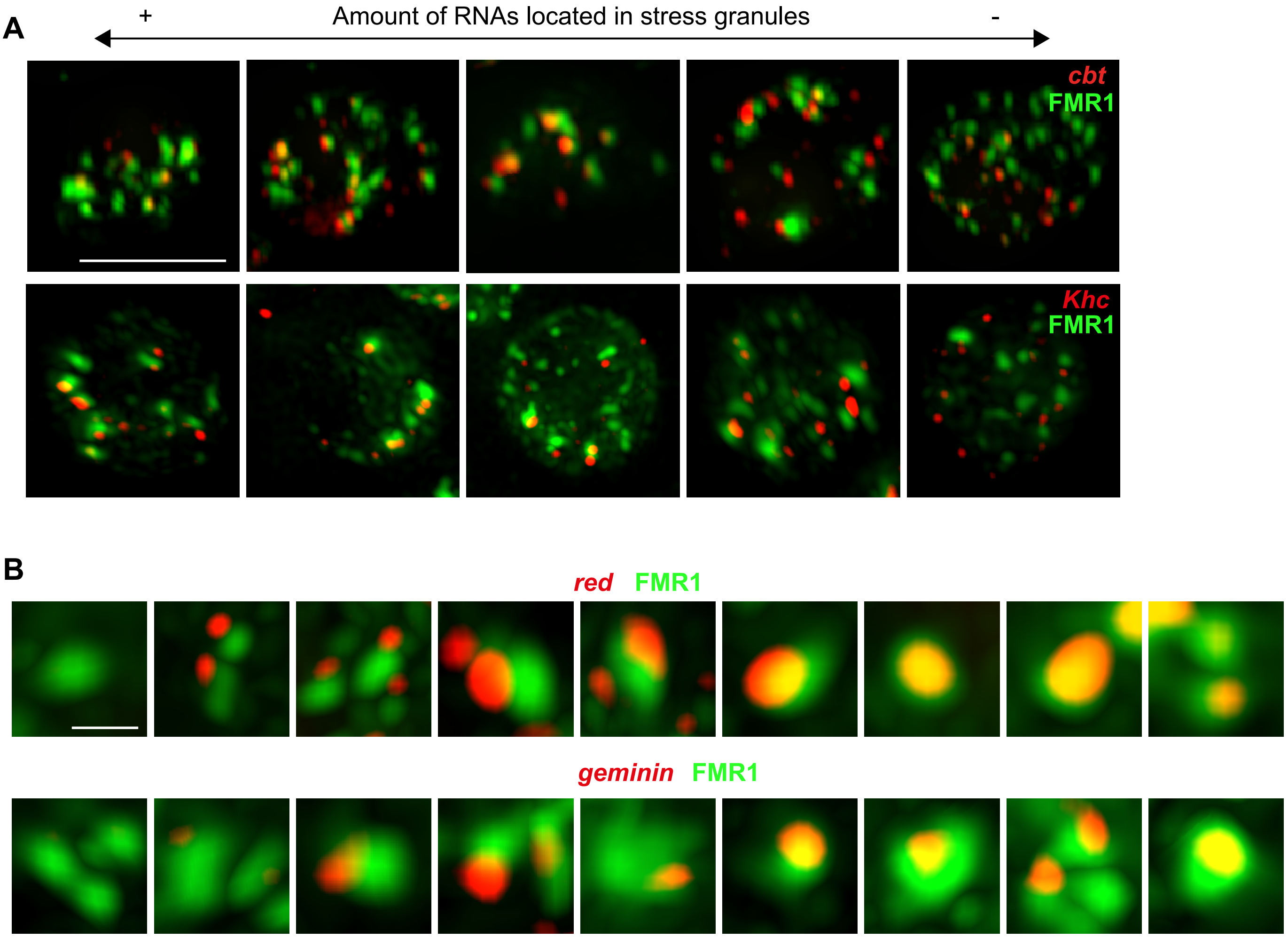

Legends for Supplemental figures

***Suppl figure S1*: FMR1 intensity in stress granules and cell survival on arsenite stress, related to Figure 1.**

A: Measurement of the fluorescence intensity of FMR1-ADARcd-V5 in stress granules when compared to the cytosolic fraction. Quantification of the ratio (peaks vs dip) in 10 cells/30 granules (A').

B: Graph displaying the survival of S2 cells after arsenite treatment (0.5 mM, 4 h) followed by recovery in Schneider's up to 5 days (168 h). Note that 20% of S2 cells die after the arsenite treatment and start growing again after 48 h at a similar rate as their non-treated counterparts.

C: Graph displaying the dissolution of stress granules in S2 cells after arsenite treatment (0.5 mM, 4h) followed by recovery in Schneider's from 3 to 168 h.

***Suppl figure S2*: Frequency of other possible base changes, related to Figure 1.**

A-C: Graph displaying the frequency of G>T, T>C or G>A editing events in S2 cells in which the expression of FMR1-ADARcd-V5 was induced for 20 min and 4 h. Note that there were no editing events for these combinations.

***Suppl figure S3*: Correlation RNA expression levels, related to Figure 2.**

A: Heatmap displaying the correlation of RNA expression level between the triplicates of S2 cells.

B: Heatmap displaying the correlation of RNA expression level between the edited transcriptome of S2 cells.

***Suppl figure S4*: FMR1-ADARcd edits transcripts at select sites, related to Figure 2, Figure 7 and STAR methods.**

A: Mutation rates at each base along a 500 nt region of the *pzg* transcript. A to G transitions are indicated in red and all other mutation classes in dark grey. The variant sites used to quantify the edit rates per transcript are indicated by light grey vertical lines and are the same in each sample type and treatment. Note that the mutation rates for the A to G position are higher in the induced samples (Schneider's and arsenite).

B: Quantification of the observation (made in A) in a genome-wide manner. This further demonstrates that FMR1-ADARcd produces rare, moderately-edited sites. For example, when we compared the distributions of the A to G mutation frequencies on all exonic bases covered by more than 10 reads between the uninduced (green) and induced (blue) and arsenite stressed (red) conditions in S2 cells (left), there were several notable observations. First, the majority of bases are not mutated in any conditions (leftmost bin). Next, certain bases with nearly saturated mutation frequencies are equally abundant in the different treatments (rightmost bins, A to G mutation frequency > 0.9). Finally, a substantial increase in the number of bases with moderate edit frequencies (e.g., approximately between 0.1 and 0.9) is observed. While not as striking, these same behaviors were also seen in the dissociated brain samples. Thus, FMR1-ADARcd produces edits at rare select bases.

However, despite this substantial increase in abundance of moderately-edited bases in the induced and stress conditions, the edited bases are rare. Based on these observations on the nature of the editing signal, variants were identified using these variant positions to quantify editing frequencies. Thus, substantially reducing the influence from other mutational processes (such as sequencing and amplification errors) in our quantification of editing frequencies. Additionally, to reduce the potential influence of genomic variants, we do not consider any positions that are known variants (Ensembl version 95) or those with a mutation frequency greater than 0.95.

C: Data processing workflow and example of gene-wise mutation frequency calculation. For each detected A to G variant position, the editing frequency was calculated across all conditions. Per condition all the editing frequencies along a transcript were averaged to get the average editing frequency.

Suppl figure S5**: smFISH depicting the heterogeneity between cells and stress granules, related to Figure 3**

A: Visualization of *cbt* and *Khc* RNAs by smFISH in S2 cells (red) showing the heterogeneity in the amount of RNA molecules located in stress granules (FMR1) between different S2 cells. Cells on the far left have stress granules with more RNA molecules than cells on the far right. This suggests a heterogeneity between cells.

B: Visualization of *red* and *geminin* mRNAs by smFISH showing that stress granules (marked by FMR1, red) can be heterogenous in RNA content within cells. Stress granules on the far left do not contain the tested RNA molecules, while stress granules on the far right contain many of tested RNA molecules. This suggests a heterogeneity between stress granules within a cell.

Scale bar: 10 µm (A), 1 µm (B).
